# An optimization framework for targeted spinal cord stimulation

**DOI:** 10.1088/1741-2552/acf522

**Published:** 2023-09-28

**Authors:** Ehsan Mirzakhalili, Evan R Rogers, Scott F Lempka

**Affiliations:** 1 Department of Biomedical Engineering, University of Michigan, Ann Arbor, MI, United States of America; 2 Biointerfaces Institute, University of Michigan, Ann Arbor, MI, United States of America; 3 Department of Anesthesiology, University of Michigan, Ann Arbor, MI, United States of America

**Keywords:** spinal cord stimulation, neurostimulation, neuromodulation, computer modeling, finite element modeling, chronic pain, optimization

## Abstract

*Objective*. Spinal cord stimulation (SCS) is a common neurostimulation therapy to manage chronic pain. Technological advances have produced new neurostimulation systems with expanded capabilities in an attempt to improve the clinical outcomes associated with SCS. However, these expanded capabilities have dramatically increased the number of possible stimulation parameters and made it intractable to efficiently explore this large parameter space within the context of standard clinical programming procedures. Therefore, in this study, we developed an optimization approach to define the optimal current amplitudes or fractions across individual contacts in an SCS electrode array(s). *Approach*. We developed an analytic method using the Lagrange multiplier method along with smoothing approximations. To test our optimization framework, we used a hybrid computational modeling approach that consisted of a finite element method model and multi-compartment models of axons and cells within the spinal cord. Moreover, we extended our approach to multi-objective optimization to explore the trade-off between activating regions of interest (ROIs) and regions of avoidance (ROAs). *Main results*. For simple ROIs, our framework suggested optimized configurations that resembled simple bipolar configurations. However, when we considered multi-objective optimization, our framework suggested nontrivial stimulation configurations that could be selected from Pareto fronts to target multiple ROIs or avoid ROAs. *Significance*. We developed an optimization framework for targeted SCS. Our method is analytic, which allows for the fast calculation of optimal solutions. For the first time, we provided a multi-objective approach for selective SCS. Through this approach, we were able to show that novel configurations can provide neural recruitment profiles that are not possible with conventional stimulation configurations (e.g. bipolar stimulation). Most importantly, once integrated with computational models that account for sources of interpatient variability (e.g. anatomy, electrode placement), our optimization framework can be utilized to provide stimulation settings tailored to the needs of individual patients.

## 1. Introduction

Spinal cord stimulation (SCS) is a neurostimulation approach to manage chronic pain in patients who do not respond to conventional treatments. More than 50 000 units are implanted annually [1], and the demand for SCS is only expected to increase as a non-addictive alternative to opioids [2]. Yet, the clinical success of SCS is limited and only about 58% of patients receive adequate long-term pain relief [3].

To overcome these shortcomings, new technologies have been developed to expand stimulation paradigms not only to decrease the number of non-responders but to also improve the benefits (maximum pain relief with minimum side effects) of SCS [4]. However, more options have not readily translated to better clinical outcomes [5] because the mechanisms of action for SCS remain elusive [6] and finding optimal stimulation parameters continues to be challenging [7].

The common practice for programming neurostimulation devices is mostly a trial-and-error process [8], which is time consuming, subject to inter-operator variability, and prone to failure [9, 10]. Moreover, the available stimulation settings (>10^16^ permutations) are only expected to increase as systems with advanced waveform capabilities and high-density electrode arrays continue to be researched and commercialized [11]. Therefore, the current practice of trial-and-error programming is becoming intractable. Hence, a systematic paradigm that can efficiently explore the large parameter space and suggest efficacious stimulation settings can improve clinical decision support [12]. In this study, we developed a novel optimization framework to facilitate SCS programming.

**Figure 1.**
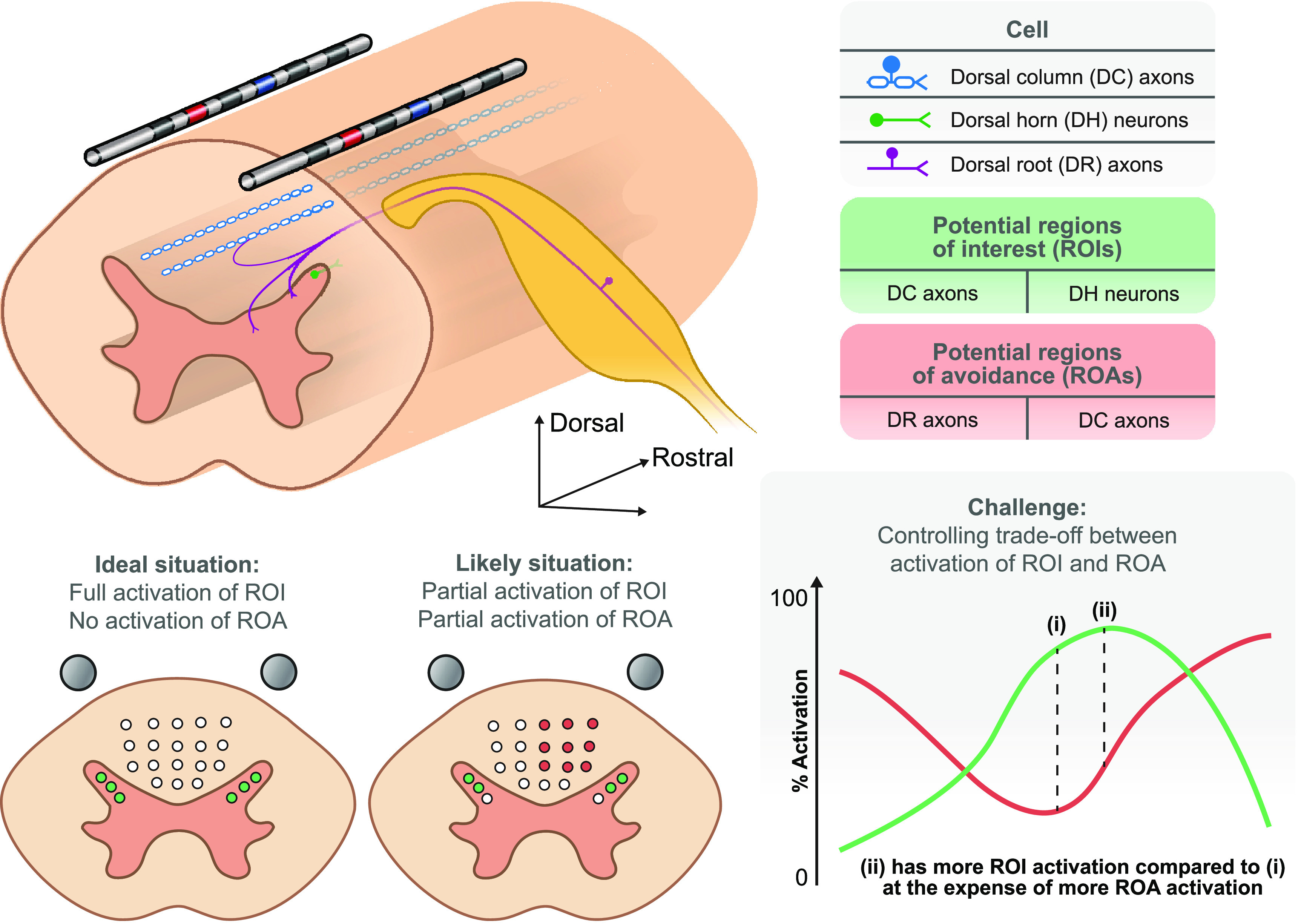
Targeted spinal cord stimulation (SCS) requires optimization of electrode current fractions. SCS affects each cell type differently due to many factors including their intrinsic cellular properties, location relative to the electrodes, and their morphology. The recruited cells can have beneficial effects if they are located in a region of interest (ROI), or they can have side effects if they are located in a region of avoidance (ROA). Ideally, SCS would activate cells in the ROI (e.g. dorsal horn (DH) neurons) without activating cells in the ROA (e.g. dorsal root (DR) axons). However, in most situations, cells in both the ROI and ROA may be simultaneously activated. Hence, the challenge is to systematically find electrode configurations that allow for controlling the trade-off between activating ROIs and ROAs.

Contrary to our optimization approach, previous studies assume a desired potential field and derive electrode current fractions that produce the best match to the desired field [12]. Instead, in our approach, we choose the region of interest (ROI), and the algorithm finds the current fractions that lead to activation of neural tissue in the ROI by maximizing the field driving polarization of the targeted neurons (figure 1). Hence, our approach allows us to find novel potential fields that can selectively target different types of cells based on their location and morphology. Moreover, we extended our optimization approach to a multi-objective optimization framework, which allows activating neural tissues in the ROI while minimizing activation in a region of avoidance (ROA) that can be associated with side effects (figure 1). Hence, in an example application, we were able to investigate the possibility of claims that novel fields can directly target neurons in the dorsal horn (DH) (ROI) without activating axons in the dorsal column (DC) (ROA) [13].

Our results demonstrate that our optimization approach can provide novel fields to target the desired ROI and limit activation in the ROAs. In conjunction with the power of patient-specific models [14], our optimization framework can be utilized to improve the selectivity and corresponding efficacy of SCS. Finally, our optimization method is not limited to SCS and can be applied to other neurostimulation techniques, such as deep brain stimulation (DBS).

## 2. Methods

### 2.1. Single-objective optimization

The single-objective optimization problem consists of maximizing the maximum of the field $\left( \mathcal{F} \right)$ that leads to the activation of neurons in the ROI:

\begin{align*}{\text{maximize}}:\max \left( {\mathcal{F}\left( {\boldsymbol{{X}}} \right)} \right){\text{ }}{\boldsymbol{{X}}} \in {\text{ROI}},{\text{ }}\end{align*}

where ${\boldsymbol{{X}}}$ represents the spatial points in the ROI. The field $\left( \mathcal{F} \right)$ can be estimated as the first derivative of the electric potential $\left( {\mathcal{F} = - {\text{d}}V/{\text{d}}r} \right)$ for terminal excitation [15–17] or the second derivative of the electric potential $\left( {\mathcal{F} = {{\text{d}}^2}V/{\text{d}}{r^2}} \right)$ for axonal excitation [18] where $\left( r \right)$ is the direction parallel to the target structure. The second derivative of the potential field is also known as the activating function and it has been widely used in neurostimulation applications [18, 19]. Regarding the rationale for equation (1), we are assuming that an action potential is initiated at a single site and propagates through the rest of the cell. Therefore, it is sufficient to formulate the optimization problem such that the maximum of the field (regardless of its location) is maximized. However, it is important to note that it is possible to consider other ways to formulate objective functions for neuromodulation. For example, it is often desired to maximize the volume of activation within a ROI [20]. To that end, instead of maximizing the maximal of the field, the integral of the field that passes a threshold would need to be maximized. While this approach requires prior knowledge to specify the appropriate threshold, this type of objective function could also be implemented within our optimization framework as described below.

To solve the optimization problem, we need to evaluate $\mathcal{F}$ as a function of the current at each electrode. Assuming quasi-static conditions [21], we can use the superposition principle to evaluate $\mathcal{F}$:

\begin{align*}\mathcal{F}\left( {{\boldsymbol{{X}}},{ }{\boldsymbol{{\alpha }}}} \right) = \mathop \sum \limits_{i = 1}^n {\alpha _i}{{\mathscr{f}}_i}\left( {\boldsymbol{{X}}} \right),{\text{ }}\end{align*}

where ${\alpha _i}$ is the current fraction at the $i{\text{th}}$ contact, $n$ is the total number of contacts, and (${{\mathscr{f}}_i}({\boldsymbol{{X}}}$) is the field obtained from the solution of the finite element method (FEM) model when the current at the $i{\text{th}}$ contact is equal to a unit current of $1{ }A$ while the current at all other contacts are $0{ }A$.

Without any constraints, the solution of equation (1) is not bounded because increasing ${\boldsymbol{{\alpha }}}$ leads to the increase in $\mathcal{F}.$ Therefore, we consider ${\boldsymbol{{\alpha }}}$ to represent current fractions rather than the actual currents (i.e. ${\boldsymbol{{\alpha }}}$ varies between −1 and +1). Indeed, adjusting current fractions is an approach that is utilized in some commercial clinical neurostimulation systems [11, 12, 22]. We also assume that the total inward (${\boldsymbol{{\alpha }}} &lt; 0)$ and outward (${\boldsymbol{{\alpha }}} &gt; 0){ }$ currents are balanced:

\begin{align*}\mathop \sum \limits_i^n {\alpha _i} = + 1\,\,\,\,\,if\,\,{\alpha _i} &gt; 0.\end{align*}

\begin{align*}\mathop \sum \limits_i^n {\alpha _i} = - 1\,\,\,\,\,if\,\,{\alpha _i} &lt; 0.\end{align*}

**Figure 2.**
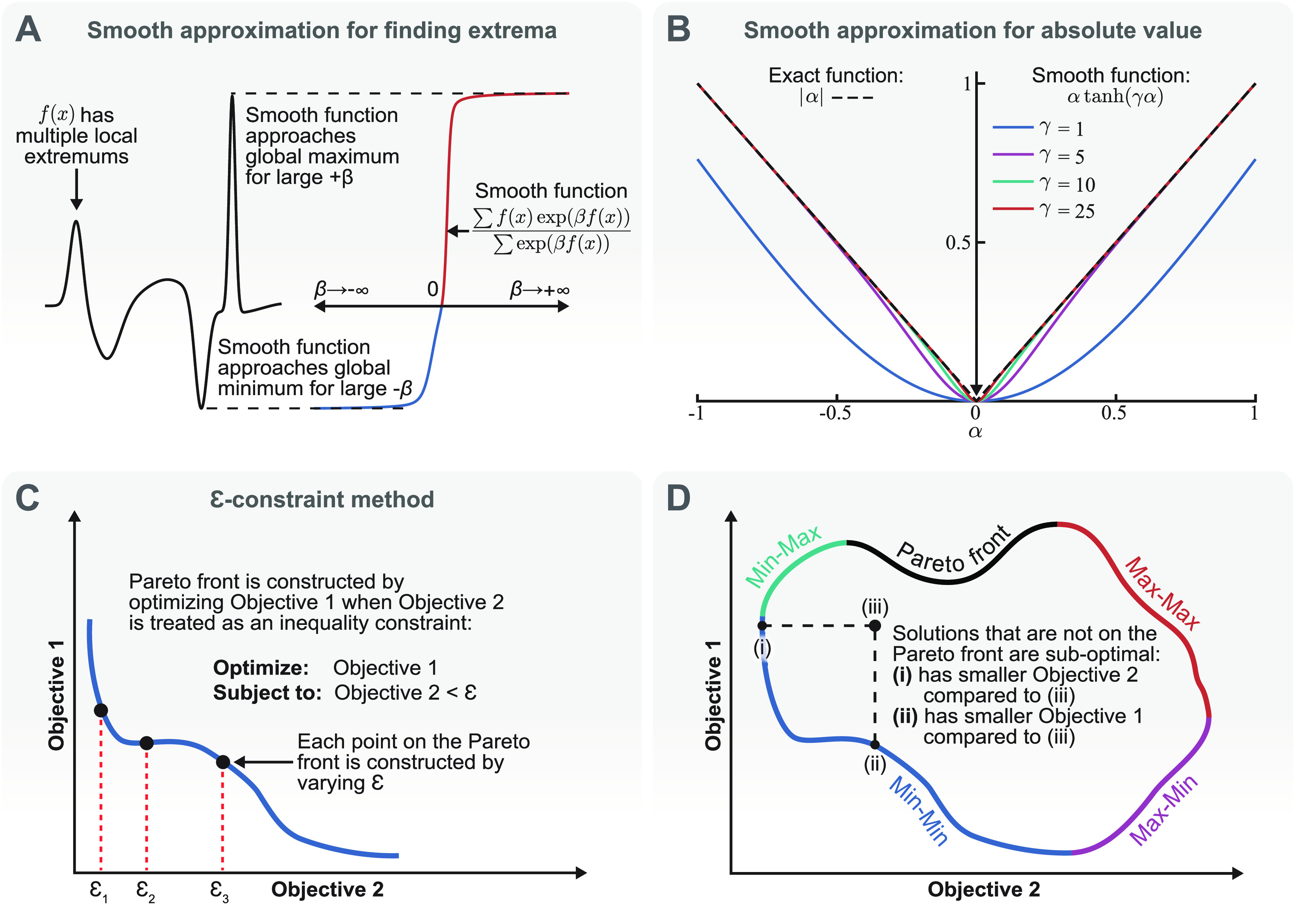
Mathematical method overview. (A) The smooth maximum function (equation (5)) can accurately approximate the largest positive value of a field (*β* > 0) or the largest negative value of a field (*β* < 0). (B) The smooth absolute value function (equation (9)) can accurately approximate the absolute value of the current fractions (*α*) and is differentiable at *α* = 0 even for large values of *γ*. (C) We use the *ε*-constraint method to construct the Pareto fronts of multi-objective optimization problems. One objective is considered as the primary objective and the other objectives are considered as inequality constraints. Then, the Pareto front is constructed by varying the value of the inequality constraints. (D) An example Pareto front for an optimization with two objectives. The points that are not on the Pareto front are sub-optimal compared to the points on the Pareto front. Multi-objective optimization for neurostimulation can consist of maximizing the objectives in all ROIs (Max–Max branch) or maximizing the objective in an ROI while minimizing the objective in an ROA (Min–Max or Max–Min branches).

Therefore, the single-objective optimization problem consists of solving equation (1) along with equations (3) and (4) as constraints. Solving this optimization problem can be challenging because the maximum operator in equation (1) is not differentiable, which prevents using analytical optimization methods, as do the conditional statements in equations (3) and (4). To overcome this challenge with regards to the objective function, we can replace the maximum operator with a smooth function that approximates the maximum of the field:

\begin{align*}&amp; {\text{ma}}{{\text{x}}_{\text{S}}}\left( {\mathcal{F}\left( {{\boldsymbol{{X}}},{\boldsymbol{{\alpha }}}} \right),\beta } \right)\nonumber\\ &amp; \quad = \frac{{\mathop \sum \nolimits_{i = 1}^m \mathcal{F}\left( {{{\boldsymbol{{X}}}_i},{ }{\boldsymbol{{\alpha }}}} \right)\exp \left( {\beta \mathcal{F}\left( {{{\boldsymbol{{X}}}_i},{ }{\boldsymbol{{\alpha }}}} \right)} \right)}}{{\mathop \sum \nolimits_{i = 1}^m \exp \left( {\beta \mathcal{F}\left( {{{\boldsymbol{{X}}}_i},{ }{\boldsymbol{{\alpha }}}} \right)} \right)}},{\text{ }}\end{align*}

where $m$ is the number of spatial points in the ROI, and $\beta &gt; 0$ is a scaling parameter. For sufficiently large $\beta $, equation (5) can approximate the maximum of the field in the ROI. In fact, equation (5) recovers the exact maximum for $\beta \to + \infty $:

\begin{align*}&amp; \mathop {\lim }\limits_{\beta \to + \infty } {\text{ma}}{{\text{x}}_{\text{S}}}\left( {\mathcal{F}\left( {{\boldsymbol{{X}}},{ }{\boldsymbol{{\alpha }}}} \right),\beta } \right)\nonumber\\ &amp; \quad = \frac{{\mathcal{F}\left( {{{\boldsymbol{{X}}}_j},{ }{\boldsymbol{{\alpha }}}} \right)\exp \left( {\beta \mathcal{F}\left( {{{\boldsymbol{{X}}}_j},{ }{\boldsymbol{{\alpha }}}} \right)} \right)}}{{\exp \left( {\beta \mathcal{F}\left( {{{\boldsymbol{{X}}}_j},{ }{\boldsymbol{{\alpha }}}} \right)} \right)}} = \mathcal{F}\left( {{{\boldsymbol{{X}}}_j},{ }{\boldsymbol{{\alpha }}}} \right),{ }\end{align*}

where $j$ is the index of the spatial point in which $\mathcal{F}$ is maximum. In practice, $\beta $ does not need to go to infinity, and as long as $\beta \mathcal{F}\left( {{{\boldsymbol{{X}}}_j},{ }{\boldsymbol{{\alpha }}}} \right)$ is sufficiently large (e.g. $\beta \mathcal{F}\left( {{{\boldsymbol{{X}}}_j},{ }{\boldsymbol{{\alpha }}}} \right) \approx 10)$, then equation (5) can provide a smooth approximation to the maximum operator. Figure 2(A) demonstrates the validity of using equation (5) to find the maximum value of a function with multiple peaks when a large enough $\beta $ is chosen. Moreover, choosing $\beta &lt; 0$ allows us to find the largest negative value of the function. As mentioned in the first paragraph of this section, it could be desirable to choose a threshold-based objective function to maximize activation within an ROI. This threshold-based objective function would require finding all the points in space that pass a certain threshold and maximizing the integral of the field that passes the specified threshold. This threshold-based objective function would also not be differentiable. However, a smooth approximation of regions that the field is less or more than the threshold could be achieved using a sigmoid function or similar. This smooth approximation would return near unity for regions that are larger than the threshold and near zero for regions that are smaller than the threshold and the transition between them could be controlled using a scaling parameter.

To make the conditional statements for the constraints (equations (3) and (4)) differentiable, we sum equations (3) and (4):

\begin{align*}\mathop \sum \limits_i^n {\alpha _i} = 0.\end{align*}

Equation (7) plainly represents the constraint that the currents need to be balanced. The information that the current fractions are between +1 and −1 is lost when we sum equations (3) and (4). However, we can first calculate the absolute values of equations (3) and (4) and then sum them together:

\begin{align*}\mathop \sum \limits_i^n \left| {{\alpha _i}} \right| = 2.\end{align*}

Equation (7) is well-posed and allows the use of analytic methods. However, equation (8) is problematic because it is not differentiable at 0, but we can replace the absolute value function with a smooth approximation:

\begin{align*}{\text{ab}}{{\text{s}}_{\text{S}}}\left( {{{\alpha }},{{\gamma }}} \right) = {{\alpha }}\tanh \left( {\gamma \alpha } \right),\,\,\end{align*}

where $\gamma &gt; 0$ is a scaling parameter. Figure 2(B) demonstrates that the absolute value function can be adequately approximated using equation (9) for large enough $\gamma $ values. Hence, we approximate equation (8) as:

\begin{align*}\mathop \sum \limits_i^n {\alpha _i}\tanh \left( {\gamma {\alpha _i}} \right) = 2.\end{align*}

Replacing the maximum operator and the absolute value function with their smooth approximations (equations (5) and (10), respectively) allows us to use the Lagrange multipliers method [23] to solve the optimization problem. To that end, we formulate the single-objective optimization problem as:

\begin{align*} {\text{maximize:}}\;{\text{ ma}}{{\text{x}}_{\text{S}}}&amp; \left( {\mathcal{F}\left( {{\boldsymbol{{X}}},{\boldsymbol{{\alpha }}}} \right),\beta } \right)\,\,\,\,{\boldsymbol{{X}}} \in {\text{ROI}} \nonumber\\ {\text{subject to:}}{ }\;{{\unicode{x1D4BD}}_1}\left( {\boldsymbol{{\alpha }}} \right)&amp; = 2 - \mathop \sum \limits_{i = 1}^n {\alpha _i}\tanh \left( {\gamma {\alpha _i}} \right) = 0 \nonumber \\ \,{\text{and}}\,\,\,{{\unicode{x1D4BD}}_2}\left( {\boldsymbol{{\alpha }}} \right)&amp; = \mathop \sum \limits_{i = 1}^n {\alpha _i} = 0,{ }\,\,\, \end{align*}

where ${\unicode{x1D4BD}}$ represents the equality constraints. Next, we can write the Lagrangian function as:

\begin{align*}\mathcal{L}\left( {{\boldsymbol{{\alpha }}},{\boldsymbol{{\lambda }}}} \right) = {\text{ma}}{{\text{x}}_{\text{S}}}\left( {\mathcal{F}\left( {{\boldsymbol{{X}}},{\boldsymbol{{\alpha }}}} \right),\beta } \right) - \mathop \sum \limits_{i = 1}^2 {\lambda _i}{{\unicode{x1D4BD}}_i}\left( {\boldsymbol{{\alpha }}} \right),\,\,\,\,\,\,\,\,\end{align*}

where ${\boldsymbol{{\lambda }}}$ is the Lagrange multiplier for the equality constraints. We can find the critical points of the Lagrangian function by taking its gradient and setting it equal to 0:

\begin{align*}{\nabla _{{\mathbf{\alpha }},{\mathbf{\lambda }}}}\mathcal{L}\left( {{\boldsymbol{{\alpha }}},{\boldsymbol{{\lambda }}}} \right) = \boldsymbol{0}.\,\,\end{align*}

Equation (13) yields a system of $n + 2$ equations ($n$ current fractions and 2 equality constraints). We solve this system of equations to find the critical points of the Lagrangian function. Finally, we use the sequence of minors of the bordered Hessian matrix to determine if these critical points are maximums, minimums, or none (saddle points) [24].

### 2.2. Multi-objective optimization

We can extend our single-optimization framework to solve multi-objective optimization problems. The most common method of solving multi-objective optimization problems is to combine the objectives into a single objective as a weighted sum. However, this simplistic approach does not allow for exploring the trade-off between different objectives, which leads to construction of the Pareto front (figure 2(D)). In our approach, we consider two types of multi-objective optimizations: (a) maximizing the fields that leads to excitation in more than one ROI (Max-Max branch in figure 2(D)), and (b) maximizing the fields that leads to excitation in the ROIs and minimizing the fields that leads to excitation in the ROAs (Min–Max and Max–Min branches in figure 2(D)). It is important to note that the field that leads to activation can be different in each ROI and ROA (e.g. first or second derivatives of the electric potential, as well as different directions).

In the case of maximizing different fields in the ROIs, the objective function in each ROI is similar to equation (1). However, the objective for minimizing the excitation fields in ROAs is:

\begin{align*}{\text{minimize}}:{\text{max }}\left( {\mathcal{F}\left( {\boldsymbol{{X}}} \right)} \right)\,\,\,\,\,\,\,{\boldsymbol{{X}}} \in {\text{ROA}}.\end{align*}

The weighted average method can be expanded to construct the Pareto front by varying the weight of each objective. However, this crude approach is prone to failure for multiple reasons (e.g. if the Pareto front is nonconvex or the objectives are not properly scaled) [25]. Instead, we use another approach known as the $ \in $-constraint method [25]. In this approach, a single objective is considered as the primary objective and all the other objectives are considered as inequality constraints:

\begin{align*}\begin{gathered} {\text{maximize:}}\;{{\max} _{\text{S}}}\left( {{\mathcal{F}_1}\left( {{\boldsymbol{{X}}},{\boldsymbol{{\alpha }}}} \right),\beta } \right)\,\,\,\,\,{\boldsymbol{{X}}} \in {\text{ROI}} \hfill \\ {\text{subject to:}}\;{{\unicode{x210A}}_i} = {{\max} _{\text{S}}}\left( {{\mathcal{F}_i}\left( {{\boldsymbol{{X}}},{\boldsymbol{{\alpha }}}} \right),\beta } \right) - { \epsilon _i} \leqslant 0 \hfill \\ \qquad \qquad\; {\text{ }}{\boldsymbol{{X}}} \in {\text{RO}}{{\text{I}}_i}{\text{ or RO}}{{\text{A}}_i}{ }\, \hfill \\ \end{gathered} \end{align*}

where $i \geqslant 2$ is the index of the $i{\text{th}}$ ROI or ROA. Then, we can vary $ \epsilon $ (figure 2(C)) to construct the Pareto fronts even if they are not convex [25].

We use the generalized Lagrange multipliers method to solve the new single-objective optimization problem, which includes inequality and equality constraints (equations (7) and (10)). For a given $ \epsilon $, we write the Lagrangian expression as:

\begin{align*} \mathcal{L}\left( {{\boldsymbol{{\alpha }}},{\boldsymbol{{\lambda }}},{\boldsymbol{{\mu }}}} \right)&amp; = {\text{ma}}{{\text{x}}_{\text{S}}}\left( {{\mathcal{F}_1}\left( {{\boldsymbol{{X}}},{\boldsymbol{{\alpha }}}} \right),\beta } \right) - \mathop \sum \limits_{i = 1}^2 {\lambda _i}{{\unicode{x1D4BD}}_i}\left( {\boldsymbol{{\alpha }}} \right)\nonumber\\ &amp; \quad - \mathop \sum \limits_{i = 2}^Q {\mu _i}{{\unicode{x210A}}_i}\left( {{\boldsymbol{{\alpha }}},{ \epsilon _q}} \right),\,\,\end{align*}

where ${\boldsymbol{{\lambda }}}$ and ${\boldsymbol{{\mu }}}$ are the Karush–Kuhn–Tucker (KKT) multipliers, and $Q$ is the total number of ROIs or ROAs. We can find the critical points of the generalized Lagrangian function by using the KKT conditions:

\begin{align*}{\nabla _{{\boldsymbol{\alpha}} ,{\boldsymbol{\lambda}} ,{\boldsymbol{\mu}} }}\mathcal{L}\left( {{\boldsymbol{\alpha}} ,{\boldsymbol{\lambda}} ,{\boldsymbol{\mu}} } \right) = \boldsymbol{0}\end{align*}

\begin{align*}{\unicode{x210A}}\left( {{{\boldsymbol{{\alpha }}}^{\boldsymbol{{*}}}}, \epsilon } \right) \leqslant \boldsymbol{0}\end{align*}

\begin{align*}{\unicode{x1D4BD}}\left( {{{\boldsymbol{{\alpha }}}^{\boldsymbol{{*}}}}} \right) \leqslant \boldsymbol{0}\end{align*}

\begin{align*}{{\boldsymbol{{\mu }}}^{\boldsymbol{{*}}}} \geqslant \boldsymbol{0}\end{align*}

\begin{align*}\mathop \sum \limits_{i = 2}^Q \mu _i^*{{\unicode{x210A}}_i}\left( {{{\boldsymbol{{\alpha }}}^{\boldsymbol{{*}}}},{ \epsilon _q}} \right) = 0\,\,\,\,\end{align*}

where ${{\boldsymbol{{\alpha }}}^{\boldsymbol{{*}}}}$ and ${{\boldsymbol{{\mu }}}^{\boldsymbol{{*}}}}$ are solutions of the system of equations obtained from equation (17). The solution set is only valid if it satisfies the conditions shown in equations (18)–(21). Next, we use the sequence of minors of the bordered Hessian matrix to determine if the valid critical points are maximums, minimums, or none (saddle points) [24].

### 2.3. Optimization implementation

Heuristic optimization approaches rely on evaluating the objective functions and utilize different methods (e.g. evolutionary algorithms and swarm intelligence) to search for optimized solutions that also satisfy the constraints if present. The results obtained from such algorithms are not necessarily the true optimum solution of the optimization problem and should be regarded as approximate solutions. Furthermore, these approximate solutions are not always the global optimum, and the search algorithm might have only found a local optimum solution.

However, the Lagrange multiplier method provides a set of equations that by solving them provides the critical solutions of the objective functions. The type of critical point (minimum, maximum, or none) is determined using exact mathematical techniques (bordered Hessian matrix [24]), which does not require evaluating the objective function itself. However, the objective functions can be evaluated at these critical points to determine which one of them is the global optimum solution.

In this study, we obtained the solutions to our optimization problem by solving the set of equations obtained from equation (13) for single-objective or equation (17) for multi-objective optimization problems. These are large sets of nonlinear equations, which cannot be solved analytically. Instead, we solved these sets of nonlinear equations numerically with the fsolve function in MATLAB (MathWorks, MA) with the default parameters.

The fsolve function, like most other numerical solvers, requires an initial guess to find a solution to the system of equations. Using the same initial guess leads to the same solution. Therefore, we used Latin hypercube sampling to generate a series of different initial guesses to find a set of solutions for our optimization problems. The bounds for these initial guesses for current fractions were −1 and +1 and −10 and +10 for Lagrange multipliers. It is important to note that the fsolve function does not always return a converged solution or a solution with real values. We disregarded the imaginary solutions or the solutions that did not converge. Then, we checked the valid solutions to determine if they are maximums, minimums, or none, and only stored the optimal solutions for further evaluation (evaluating the objective functions to find the global solution or constructing Pareto fronts). It is also important to consider that using the fsolve function naively to find a solution can be time consuming and the computational time accumulates because we used fsolve for many different initial guesses. However, the fsolve function can converge much faster if the Jacobian of the system of equations is passed to it. Therefore, we calculated the Jacobian of the system of equations to increase the computational efficiency of our optimization approach.

Even though solutions to this system of equations can provide the exact solutions for the optimization problem, the fact that we solve them numerically can introduce some errors. Moreover, approximating equation (8) (the absolute value function) with equation (10) leads to solutions that do not perfectly satisfy the exact constraints (equations (3) and (4)). Therefore, we performed one additional step (equation (22)) after accepting a solution from the fsolve function to make sure that the solution satisfied the original constraints completely:

\begin{align*}{{\boldsymbol{{\alpha }}}_{{\mathbf{{corrected}}}}} = \frac{{{{2\boldsymbol{{\alpha }}}^{\boldsymbol{{*}}}}}}{{\mathop \sum \nolimits_i^n \left| {\alpha _i^*} \right|\,}},{\boldsymbol{{\,\,\,\,\,\,\,}}}\end{align*}

where ${{\boldsymbol{{\alpha }}}^{*}}$ is a valid set of optimized current fractions, ${{\boldsymbol{\alpha}}_{{\mathbf{corrected}}}}$ is the corrected set of optimized current fractions that perfectly satisfy the exact constraints (equations (3) and (4)), and $n$ is the total number of contacts. We should note that the values of corrected fractions (equation (22)) have full machine accuracy, which are not feasible in practice with clinical systems. Instead, the values of the optimized current fractions are rounded. We rounded the optimized current fractions with three significant digits for the results presented in this paper and ensured that these rounded current fractions still satisfied the exact constraints (equations (3) and (4)).

### 2.4. Illustrative example

**Figure 3.**
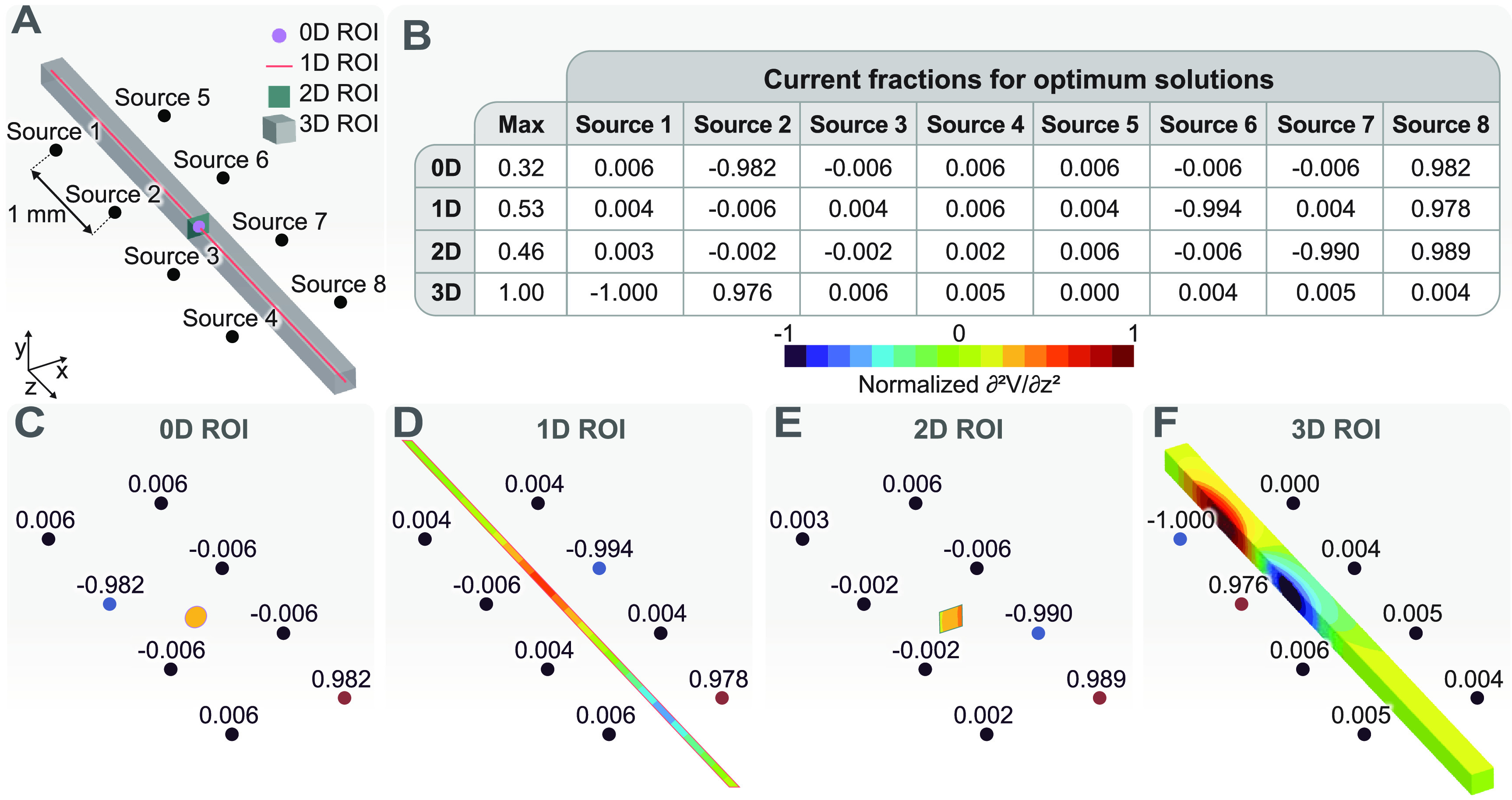
Optimization of the activating function in an ROI with various dimensions for an ideal setup. (A) The ROIs of various dimensions are placed symmetrically between eight ideal current sources. (B) The current fractions for each ROI and the maximum value of the activating function. (C)–(F) Normalized activating function in each ROI for the optimized configurations. The activating function (second derivative of the electric potential in the *z*-direction) is normalized using the largest value of the activating function across all ROIs.

Here, we demonstrate the optimization procedure using an ideal setup. Figure 3(A) shows eight current sources placed symmetrically in space. We calculated the potential field generated for each monopole current source $I$ (sources 1–8) located at (${x_s}$, ${ }{y_s}$, ${z_s}$) in the anisotropic volume as equation (23) [26]:

\begin{align*}V\left( {x,y,z} \right) = \frac{I}{{4\pi \sqrt {{\sigma _{xx}}{\sigma _{yy}}{\sigma _{zz}}} }}\frac{1}{{\sqrt {\frac{{{{\left( {x - {x_s}} \right)}^2}}}{{{\sigma _{xx}}}} + \frac{{{{\left( {y - {y_s}} \right)}^2}}}{{{\sigma _{yy}}}} + \frac{{{{\left( {z - {z_s}} \right)}^2}}}{{{\sigma _{zz}}}}} }}{\boldsymbol{{\,}}}\end{align*}

where ${\sigma _{xx}}$, ${\sigma _{yy}}$, and ${\sigma _{zz}}$ are the conductivities in the $x$, $y$, and $z$ directions, respectively. Assuming that the axons were positioned parallel to the $z$-axis, we used ${\sigma _{zz}} = 0.6{\text{ S}}\;{{\text{m}}^{ - 1}}$ and ${\sigma _{xx}} = {\sigma _{yy}} = 0.083\,\;{\text{S}}\;{{\text{m}}^{ - 1}}$ to account for higher conductivity of white matter in the longitudinal direction compared to the transverse direction [27–29].

The optimization problem for this example consists of maximizing the second derivative of the electric potential (activating function) [18] in the *z*-direction for ROIs of various dimensions. In this example, we mainly used the various dimensions for ROIs to explain the concept. However, in practice, it can be desirable to use lower dimensional ROIs/ROAs because they are computationally less expensive and can provide useful results if they are properly selected. The table in figure 3(B) shows the current fractions for the optimized configurations shown in figures 3(C)–(F). We can see that the current fractions for all ROIs satisfy the constraints of the optimization problem (equations (3) and (4)). Interestingly, the absolute values of the optimized current fractions are either close to zero or close to one. Therefore, all optimized configurations can be considered as bipolar configurations (i.e. one source acts as the cathode and the second source acts as the anode).

The dimension and location of the ROI determines which source acts as the cathode and which source acts as the anode. For the 0D ROI (single point shown in figure 3(C)), Sources 2 and 8 can be considered as the cathode and anode, respectively. The choice of these two sources to maximize the activating function for this 0D ROI is not intuitive but we should note that this solution was not unique and other combinations of cathodes and anodes provided solutions with similar values of the objective function. In general, there could exist several optimized solutions with the same objective function values for ROIs of any dimension. However, the number of such solutions decreases as the number of dimensions for the ROI increases. Sources 6 and 8 can be considered as cathode and anode, respectively, for the 1D ROI (line shown in figure 3(D)). Again, this solution is not unique, and any combination of cathodes and anodes separated by a single source would result in equally good objective functions.

We can observe that the maximum value of the objective function for the 1D ROI is larger compared to the maximum value for the 0D ROI. This result is expected because the location of the 0D ROI is not necessarily where the activating function can reach its peak. However, the 1D ROI has the possibility of including points where the activating function reaches its peak. Hence, the optimization algorithm finds a set of current fractions that produces a higher maximum value of the objective function for the 1D ROI compared to 0D ROI. In fact, a higher dimensional ROI is guaranteed to have a larger or equal objective function value compared to a lower dimensional ROI if the lower dimensional ROI is a subset of the higher dimensional ROI. The results for the 2D ROI (plane shown in figure 3(E)) and the 3D ROI (figure 3(F)) clearly demonstrate this rule. The 2D ROI has a larger objective function compared to the 0D ROI (the 0D ROI is a subset of the 2D ROI). However, there is no guarantee that the 2D ROI should have a larger objective function compared to the 1D ROI because not all points of the 1D ROI are included in the 2D ROI. In fact, the objective function for the 1D ROI is larger compared to the 2D ROI. The 2D ROI plane is not placed where the activating function can reach its peak compared to the 1D ROI. Hence, the 1D ROI can reach larger activating function values even though the 2D ROI has points closer to the sources. The 3D ROI encompasses all of the lower dimensional ROIs. Hence, it achieves the largest objective function because it has the freedom to include points that are closer to the sources (similar to the 2D ROI), but it also has the freedom to have these points at the proper *z* location.

While it might be tempting to use a large 3D volume to find the largest objective function, in practice this is not always a good approach because the largest objective function can occur somewhere away from the true ROI. For example, if the true ROI was somewhere between sources 2 and 3, then the optimization solution shown in figure 3(F) would be a minimum for the actual ROI instead of a maximum.

### 2.5. Finite element analysis

**Figure 4.**
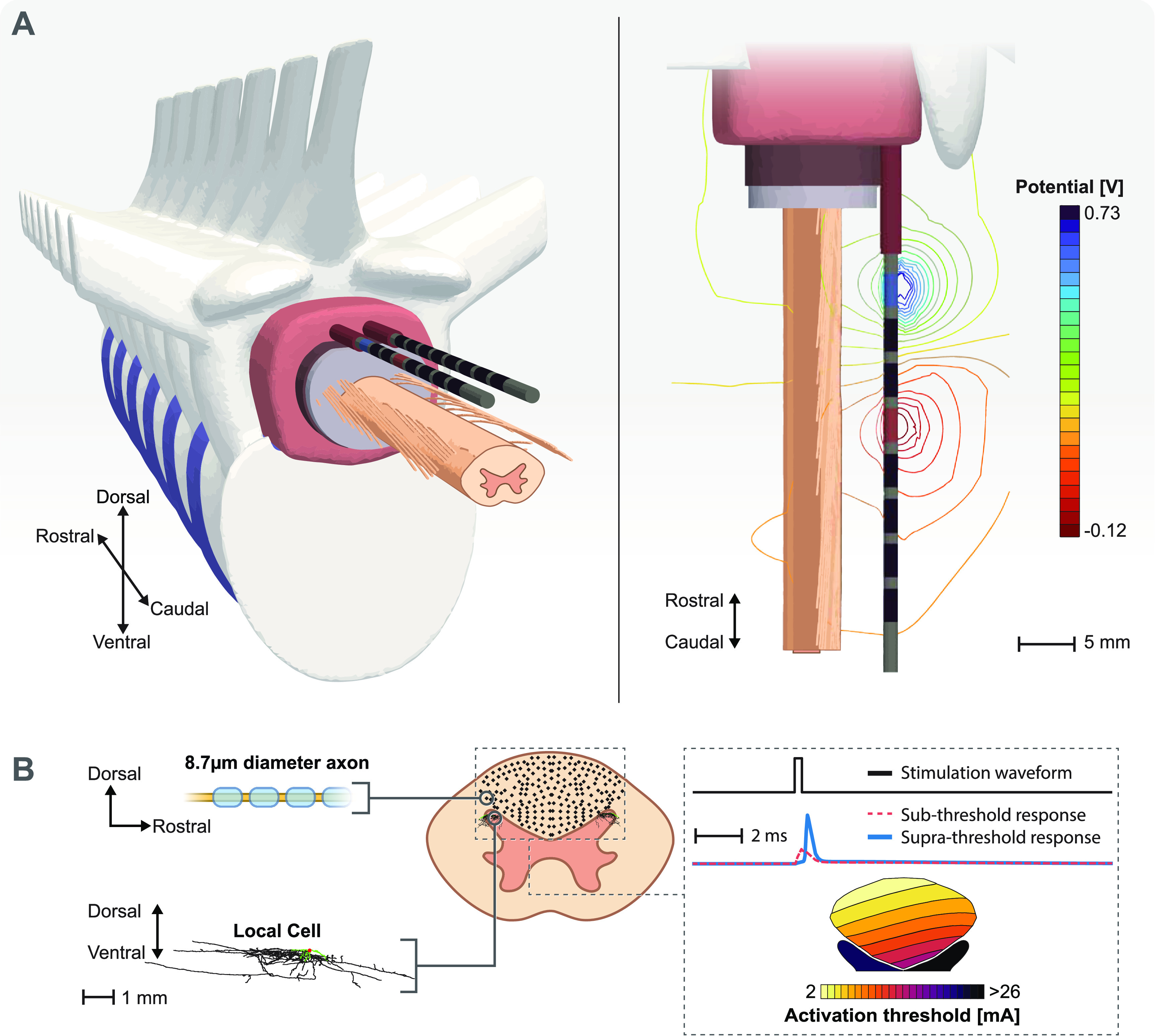
Computational model overview. (A) Exploded views of the three-dimensional canonical anatomy used in the finite element method (FEM) model. The iso-potentials generated around the stimulating electrodes as the result of unit currents applied at the anode (+1 A) and the cathode (−1 A). (B) We populated the dorsal columns with axons and dorsal horns with local cells. We integrated the FEM model with biophysical simulations to find the activation thresholds of all cells in response to spinal cord stimulation.

Previous studies have shown the importance of detailed modeling for SCS [14, 30]. Hence, we used a lower thoracic spinal cord FEM model as shown in figure 4(A) to examine the strength of our optimization algorithm for targeted SCS. This volume conductor model has been described in detail [31], which includes gray matter, white matter, cerebrospinal fluid (CSF), dura mater, epidural fat, vertebral bone, intervertebral discs, an electrode encapsulation domain, and thorax. Table 1 provides the electrical conductivity of each region. The SCS leads were two cylindrical percutaneous electrode arrays with eight contacts. The diameter of each SCS lead was 1.3 mm and the center-to-center distance between them was 4 mm. Each electrode was 3 mm long with an edge-to-edge spacing of 1 mm between each electrode.

**Table 1.** Electrical conductivities for the FEM model tissues.

Tissue	Conductivity (S m^−1^)	References
Gray matter	0.23	[32]
White matter (longitudinal)	0.60	[32]
White matter (transverse)	0.083	[32]
Cerebrospinal fluid (CSF)	1.70	[32]
Dura mater	0.60	[33]
Epidural fat	0.25	[34]
Vertebral bone	0.02	[35]
Intervertebral disc	0.65	[32]
Electrode encapsulation	0.11	[36]
Thorax	0.25	[32]

We created and meshed the volume conductor geometry using 3-matic (Materialize, Belgium). We used a higher mesh density near the electrode contacts to achieve a more accurate solution of the electric potential field. We imported the volume mesh into COMSOL Multiphysics (COMSOL, MA) to solve the Laplace equation (equation (24)) to obtain the electrostatic potential fields generated during stimulation:

\begin{align*}\nabla \cdot \sigma \nabla V = 0,\,\,\,\,\,\,\,\,\end{align*}

where $\sigma $ is the electrical conductivity and $V$ is the electric potential. We grounded ($V = 0$) the outer surface of the volume conductor model and treated each contact as floating potentials with the desired net current. To evaluate the fields (${\mathscr{f}}({\boldsymbol{{X}}}$) in equation (2) used in the optimization problem, we applied +1 A to a single electrode while the rest of the electrodes were inactive (0 A). Figure 4(A) shows an example isopotential field around the stimulating electrodes as the result of superposition for unit currents at the anode (+1 A) and the cathode (−1 A).

It is important to recognize that ${\mathscr{f}}\left( {\boldsymbol{{X}}} \right)$ can be different from the potential field ($V$) itself and depends on the type of field that can predict the excitation of cells (e.g. first derivative of the potential field for terminal excitation and the second derivative of the potential field for axonal excitation, as discussed in section 2.1). We calculated the derivatives directly in COMSOL to take full advantage of the second-order shape functions used in our FEM model.

### 2.6. Cell models

In this study, we tested the results of our optimization framework on axons located in the DCs and cells located in the DH (figure 4(B)).

#### 2.6.1. DC axons

We used a modified version of a well-established compartmental model of a myelinated mammalian axon to investigate the response of axons located in the DCs [26, 37]. This axon model (figure 4(B)) includes active nodes of Ranvier and passive internode regions. The nodes of Ranvier included fast sodium, persistent sodium, slow potassium, leakage, and capacitive currents. The modified version [26] of the model fixes issues with the original model [37] in which both the backward and forward rates for the slow potassium channel increased with the membrane voltage. We used the same morphology as the original double-cable model, which included myelin attachment segment, paranode main segment, and internode segment regions of the fiber. In this study, we modeled axons with a diameter of 8.7 *μ*m.

#### 2.6.2. DH cells

We utilized a local cell model without change as described in detail in the original publication [38], but we briefly summarize the model design here. To maximize biological realism, we used the cell morphology of a previously published reconstruction of a large lamina I interneuron (Neuromorpho ID NMO_34018 [39]). The axon was myelinated following the algorithm described by Aberra and colleagues [40], and given a specific membrane capacitance of 0.02 *μ*F cm^−2^ and specific membrane resistance of 1.125 MΩcm^2^. Nodal compartments contained fast sodium (3.45 S cm^−2^) [41] and delayed rectifier potassium (0.076 S cm^−2^) [42], in addition to a specific membrane resistivity of 91 kΩcm^2^ [43] and specific membrane capacitance of 0.85 *μ*F cm^−2^ [44]. Model neurons were validated based on comparison to relevant frequency-intensity relationships during injected current clamp experiments [45] as well as the response to extracellular microstimulation [46].

#### 2.6.3. Model implementation

To assess neural recruitment during SCS, we used the NEURON simulation software (v7.4) with the Python programming language [47, 48]. We used the FEM model and the superposition principle (equation (2)) to calculate the overall electric potential field for a desired set of current fractions. Then, we interpolated the overall electric potential onto multi-compartment cell models to find the minimum stimulation amplitude that resulted in an action potential (supra-threshold responses in figure 4(B)). We applied these voltages to the cell models using NEURON’s extracellular mechanism. We calculated the time-dependent membrane voltage for each compartment in the cell models using NEURON’s backward Euler implicit integration method with a fixed time step of 5 *μ*s. To find the activation thresholds, we used a binary search with a relative error of 0.1%. We performed this analysis for DC fibers and local cells within the DH in response to a monophasic cathodic stimulus with a pulse width of 300 *μ*s (figure 4(B)).

## 3. Results

We used our optimization framework to find stimulation configurations for single- and multi-objective optimizations for one- and three-dimensional ROIs and ROAs. For each case, we evaluated the neural recruitment for the optimized stimulation configurations.

### 3.1. Single-objective optimization

#### 3.1.1. One-dimensional ROI

**Figure 5.**
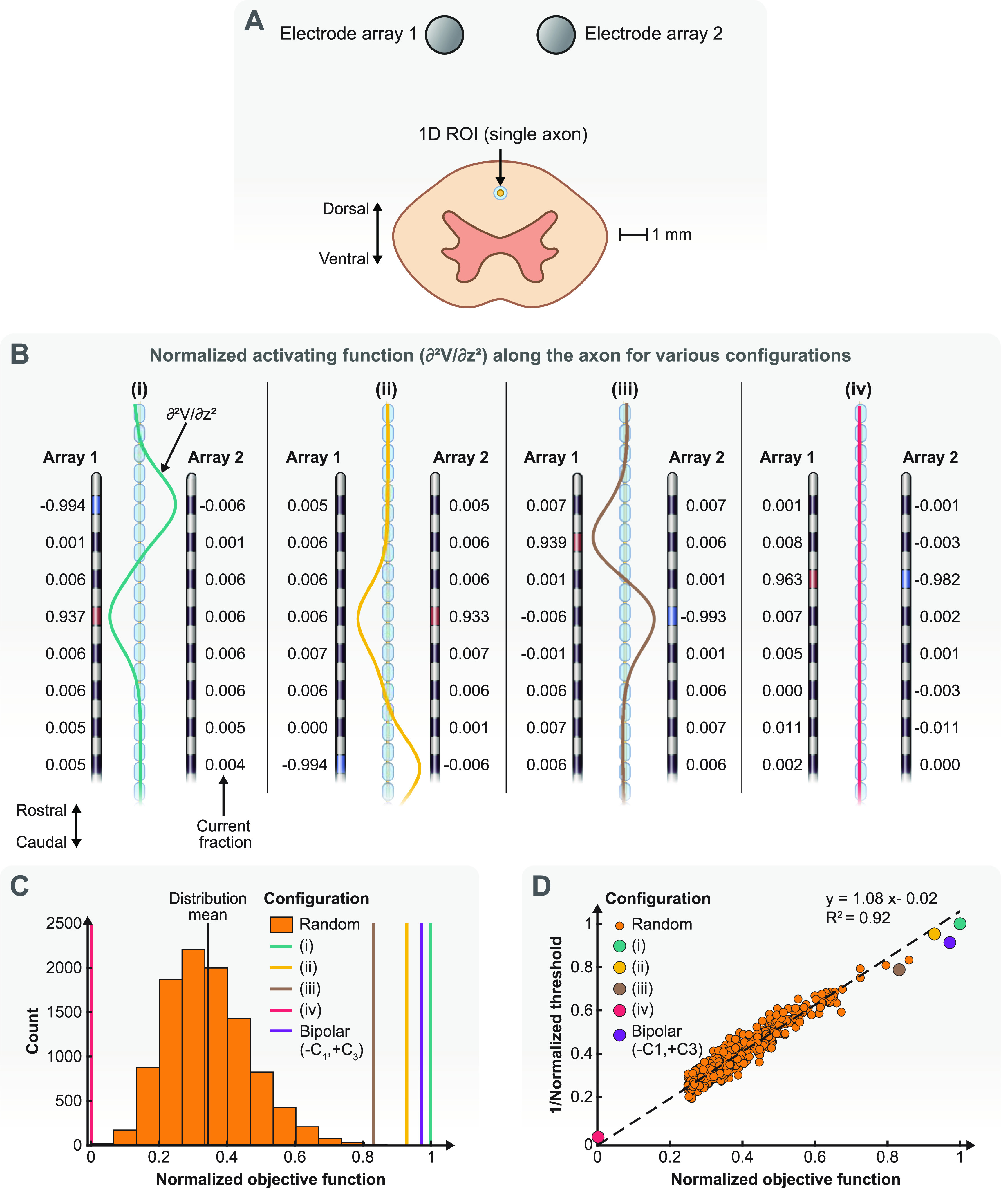
Single-objective optimization of a one-dimensional ROI. (A) The ROI is an axon in the dorsal columns that is located symmetrically between two electrode arrays. (B) Example electrode configurations obtained by our optimization algorithm. The activating function (second derivative of the potential field) is shown along the axon for each configuration. The activating functions are normalized using the peak activating function value for the configuration with the largest amplitude (configuration (i)). (C) The distribution of the normalized objective function (maximum of the activating function along the axon) for random stimulating configurations compared to the normalized objective functions obtained from our optimization algorithm. (D) The relationship between the normalized objective function (activating function) and the normalized activation threshold for the axons. Here, we only show a subset of random configurations compared to the distribution in (C) to make the figure more readable. In C and D, the objective function and activation threshold of configuration (i) are used for normalization.

The simplest ROI we used in this paper to test our proposed optimization framework for SCS was an axon located symmetrically between the two SCS electrode arrays (figure 5(A)). The objective of this optimization was to find stimulation configurations that result in the smallest activation threshold of the ROI. To that end, we maximized the activating function along the axon.

Figure 5(B) demonstrates some example configurations found by our optimization framework. We normalized all the activating functions using the peak of the configuration with the largest amplitude (configuration (i)). As we described in the section 2, the Lagrange multiplier method finds all the critical points of the Lagrangian function. These critical points can be maximums (configurations (i)–(iii)) or minimums (configuration (iv)), which can also be ranked to determine global optimum solutions. The results in figure 5(B) suggests that the largest activating functions can be achieved using only a single electrode array (configuration (i)) (i.e. array 1 includes electrodes with large current fractions, while array 2 only includes small current fractions) and only two electrodes (one cathode and one anode) are sufficient to obtain large activating functions (configuration (i)–(iii)) (i.e. the current fractions are large at two electrodes and small at all other electrodes), which is similar to the results shown in the illustrative example (figure 3). However, both electrode arrays are needed to obtain an activating function close to zero in the ROI (configuration (iv)). This configuration is meaningless for the ROI because it is equivalent to no stimulation. Yet, we provided it as a prelude for multi-objective optimization where no activation in an ROA is desirable.

Figure 5(C) illustrates why a systematic approach is necessary for selecting stimulation configurations. We generated 10 000 random configurations that satisfied the constraints for the current fractions (equations (3) and (4)). It is evident that these random configurations were not close to the optimum solutions that our optimization framework was able to find. Indeed, the objective functions of random configurations were on average 66% less than the global maximum found by our optimization algorithm. Therefore, a random search algorithm that simply evaluates objective functions and sorts them is unlikely to obtain optimal solutions. Alternatively, it is possible to perform an exhaustive grid search and loop through all possible configurations (with some degree of precision). However, this approach is impractical for a large number of electrodes.

We used NEURON to calculate the activation thresholds for the stimulating configurations from our optimization algorithm and the random configurations (similar to figure 4(B)). Figure 5(D) confirms that the activating function is a great predictor of activation for the ROI (the axon in figure 5(A)). Indeed, the largest/smallest (configuration (i)/configuration (iv)) activating functions led to the smallest/largest activation threshold. These results are consistent with previous studies [18, 49]. Hence, we used the activating function as our objective function for axonal activation for the rest of this study.

#### 3.1.2. Three-dimensional ROI

**Figure 6.**
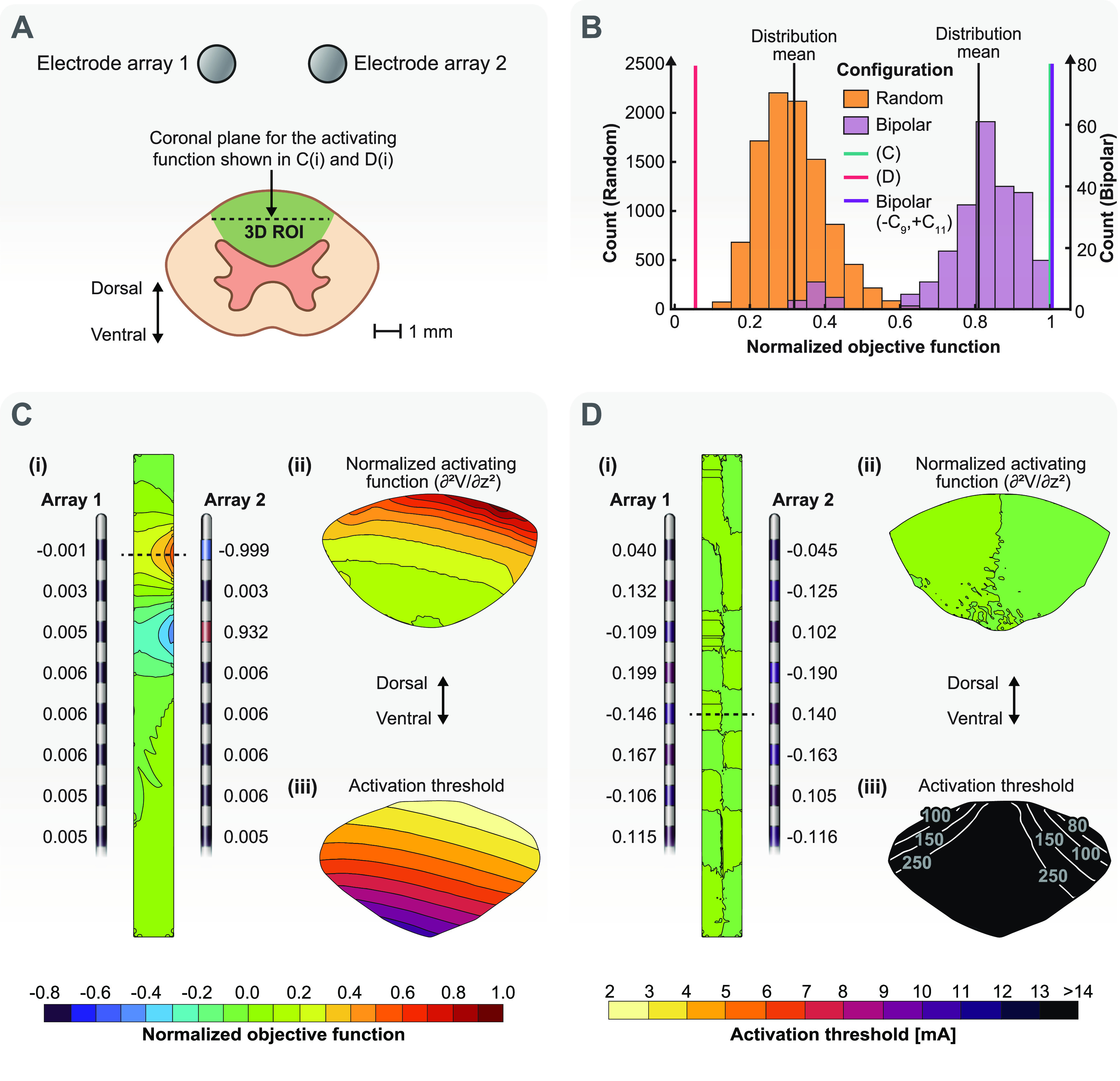
Single-objective optimization of a three-dimensional ROI. (A) The ROI is the entire dorsal columns. The goal is to maximize the activating function as the objective function. (B) The distribution of the normalized objective function (maximum of the activating function in the dorsal column) for random and bipolar stimulating configurations compared to the normalized objective functions obtained from our optimization routine. Note, the lines highlighting the normalized objective function values for the configuration shown in C and the bipolar configuration (−C_9_,+C_11_) are nearly superimposed on one another. (C) Optimization solution for the maximum objective function. (i) Stimulation configuration obtained by our optimization algorithm. The contours show the normalized activation function in the coronal plane shown by the dashed line in A. The largest value of the activating function in the ROI was used for normalization. (ii) Contours of the normalized activating function in the axial plane at the axial level shown by the dashed line in (i). (iii) Contours of the activation thresholds for axons throughout the dorsal columns (see figure 4(B)) for the stimulation configuration shown in (i). (D) Optimization solution for the minimum objective function. (i) Stimulation configuration obtained by our optimization algorithm. The contours show the normalized activation function for the coronal plane shown by the dashed line in A. (ii) Contours of the normalized activating function in the axial plane at the axial level shown by the dashed line in (i). (iii) Contours of the activation thresholds for axons throughout the dorsal columns for the stimulation configuration shown in (i).

Next, we used our optimization algorithm to find stimulation configurations when the DCs were considered as the ROI (figure 6(A)). The objective of the optimization was to find stimulation configurations that resulted in the smallest activation threshold for activating an axon anywhere within the ROI (DCs). The field to be optimized was the activating function since we were considering axonal activation.

Figure 6(B) shows that randomly finding optimum configurations is unlikely. Hence, using our optimization algorithm is beneficial even when the ROI is a large three-dimensional entity. However, considering the results shown in figure 5, we have the prior knowledge that the optimal solution is a bipolar configuration. Therefore, we can randomly evaluate the objective function for all 240 possible bipolar configurations. Indeed, the distribution mean for the bipolar configurations is larger compared to the distribution mean for the completely random configurations and some of the bipolar configurations have objective functions close to the optimized solution. In fact, a bipolar configuration (−C9, +C11) has a larger objective function compared to our optimized solution. This result is expected because the optimized configuration found by our algorithm ((i) in figure 6(C)) is equivalent to the bipolar configuration but has some small nonzero currents on other contacts due approximations and numerical issues (see section 2.3). Also, configuration (i) in figure 5(B) had a slightly smaller objective function compared to the ideal (−C1, +C4) bipolar configuration but it was still better than the objective function for the (−C1, +C3) bipolar configuration. This result highlights the importance of choosing proper cathodes and anodes even when a bipolar configuration seems to be sufficient because it is not possible to test all of the possible bipolar configurations.

The optimization algorithm for the 3D ROI can achieve a near zero objective function throughout all of the DCs (figure 6(D) (ii)), which leads to large activation thresholds for this configuration that are beyond the clinical range (e.g. 0–25 mA) [50]. Even though finding this type of configuration is meaningless for single-objective optimization, the ability to find this type of stimulation configuration is important for multi-objective optimization. For example, we can explore the possibility of recruiting cells in the DH without exciting the axons in the DCs. Moreover, figure 6(D)(i) shows that a near zero objective function is achieved when the current fraction on an electrode opposes the current fraction on the contralateral electrode at the same rostrocaudal level. It is also important to recognize that none of the random or bipolar configurations achieved a near-zero objective function.

### 3.2. Multi-objective optimization

#### 3.2.1. One-dimensional ROI and ROA

**Figure 7.**
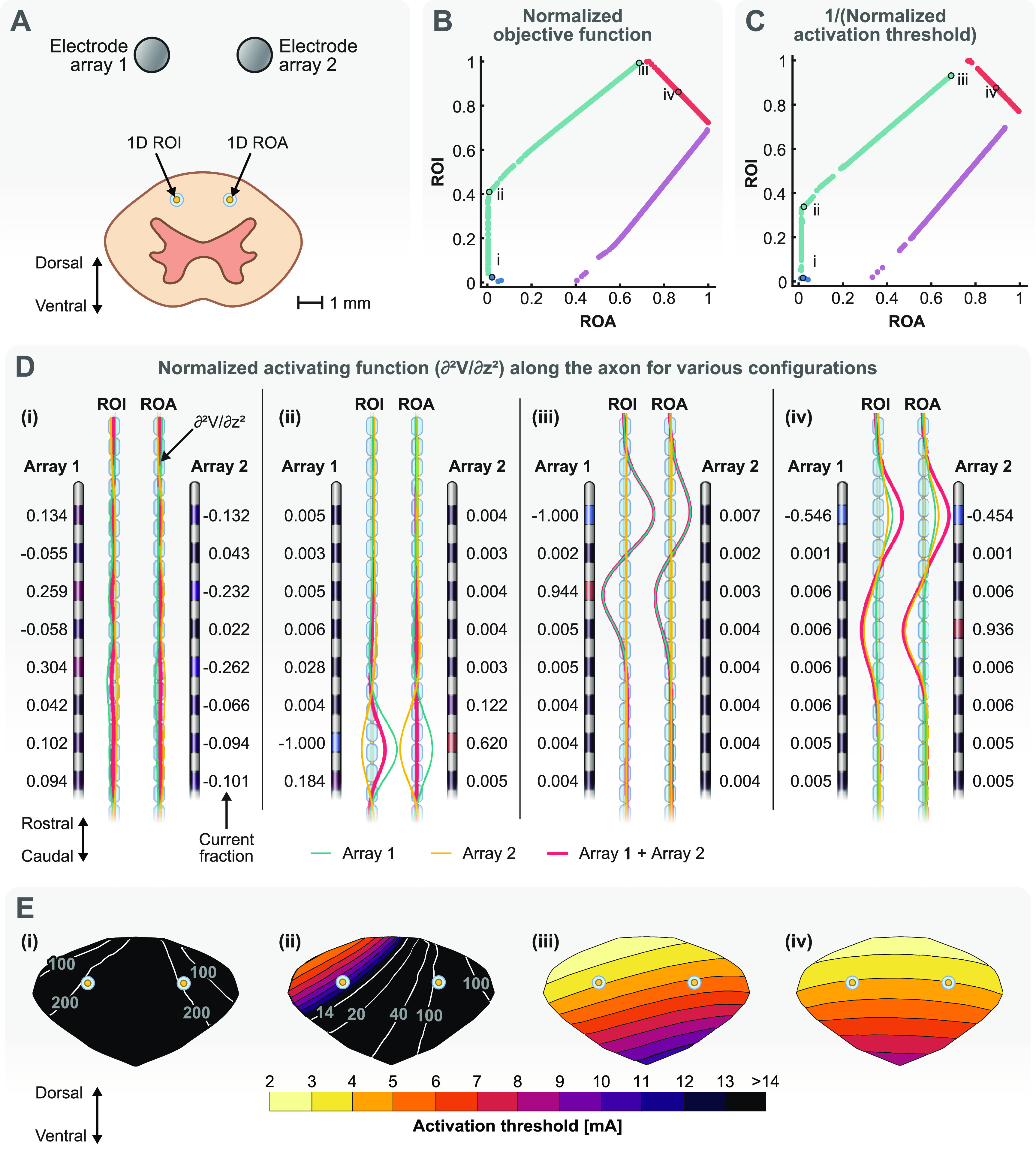
Multi-objective optimization of a one-dimensional ROI and a one-dimensional ROA. (A) The ROI is a single axon near electrode array 1 and the ROA is a single axon near electrode array 2. (B) The Pareto front of the multi-objective optimization. The objective functions (activating function) are normalized using the largest objective function detected in either the ROI or ROA. Each point on the Pareto front represents a unique stimulation configuration. We selected four points on the Pareto front for further demonstration: configuration (i) represents a case in which the objective function is small for both the ROI and ROA. Configuration (ii) represents a case in which the objective function is as large as it can get in the ROI while the objective function in the ROA is still close to zero. Configuration (iii) represents the case in which the objective function in the ROI has its largest value and greater than the objective function in the ROA. Configuration (iv) represents a case in which the objective functions in the ROI and ROA are almost equal. (C) The Pareto front for activation thresholds. The activation thresholds are normalized using the smallest activation threshold detected in either the ROI or ROA. (D) Electrode configurations and the objective function (activating function) along the ROI and ROA for the points selected in B. The objective function is normalized using the largest objective function detected in either the ROI or ROA. (E) Contours of the activation thresholds for axons throughout the dorsal columns (see figure 4(B)) for each stimulation configuration.

We used a one-dimensional ROI and a one-dimensional ROA to test our optimization framework for multi-objective optimization. The ROI and ROA are single axons that are located symmetrically in the DCs between two SCS electrode arrays (figure 7(A)). The choice of the ROI and ROA is arbitrary here, but it can have clinical justifications. For example, the programmer might want to investigate if recruiting axons on one side of the DCs versus the other side provides better targeting and subsequent pain relief for a patient. Moreover, we can consider both axons as ROIs to find configurations that provide bilateral stimulation.

Figure 7(B) shows the Pareto front for this optimization problem. The objective functions (activating function) for the ROI and ROA were normalized using the largest objective function detected. Each point on the Pareto front presents a unique stimulation configuration. We used each configuration to find the activation threshold of the ROI and ROA axons. A larger objective function (activating function) leads to a smaller activation threshold (see figure 5(D)). Hence, we calculated and plotted the Pareto front for 1/(activation threshold) (figure 7(C)), which is similar in shape to the Pareto front for the objective functions. We used the smallest activation threshold to normalize the results in figure 7(C).

Next, we selected four different configurations to demonstrate how the multi-objective optimization can be used in practice. These configurations are shown on the Pareto front of figure 7(B) (and their corresponding activation threshold in figure 7(C)). Configuration (i) represents a case where the objective function for both the ROI and ROA are small. Therefore, we found a large activation threshold (small 1/activation threshold) for them as expected (point (i) in figure 7(C)). This configuration is not useful on its own and is similar to the near zero objective function example shown for the single-objective optimization example shown in figure 6(D). However, this configuration would be meaningful if both axons were considered as ROAs and there was another location that was considered as the ROI (e.g. cells in the DH).

Configuration (ii) represents the most logical case for a two-objective optimization when there is an ROI and ROA. This point on the Pareto front was chosen because the objective function for the ROI is as large as it can get while the objective function for the ROA is still close to zero. Figure 7(D)(ii) shows that electrode arrays 1 and 2 work in opposite so that the sum of their fields is close to zero for the ROA. However, even though they are still working against each other at the ROI, they do not cancel out each other and the activating function is nonzero for the ROI. Moreover, we can see the current fractions for this configuration is not something that can be trivially selected. This result shows why it is important to have a systematic approach with parameter selection. Figure 7(E)(ii) shows that the activation threshold for the ROI and its surrounding tissue is within clinical range while the activation threshold for the ROA is beyond clinical range.

When moving from configuration (ii) to (iii) in figure 7(B), we get larger objective functions at the ROI at the expense of getting larger objective functions at the ROA. This change translates into getting lower activation thresholds at the ROI but also getting lower activation thresholds at the ROA (moving from (ii) to (iii) in figure 7(C)). In fact, configuration (iii) shows the point beyond which the objective function for the ROA starts to get larger compared to the objective function for the ROI. Hence, the Pareto front for multi-objective optimization provides a powerful tool for programmers to control the trade-off between activating ROIs at lower thresholds at the cost of activating ROAs. Figure 7(D)(iii) shows that the current fractions for this configuration are concentrated on electrode array 1. This result was expected because the ROI is closer to this electrode array, and point (iii) represents the point on the Pareto front that prioritizes activation in the ROI above anything else. Comparing (ii) and (iii) in figure 7(E) clearly shows that the consequence of activating the ROI at lower thresholds is concomitant activation of the ROA.

The red and purple branches of the Pareto fronts in figures 7(B) and (C) are not useful because they represent configurations in which ROA is activated at lower thresholds compared to the ROI. However, given the arbitrary selection of the ROI and ROA in this example, we could consider that ROA as another ROI. We could consider another example situation in which we want equal activation at both locations. Configuration (iv) provides such a non-trivial solution. We can see in figures 7(D) and (E)(iv) that the electrode arrays work together to create almost equal objective functions and corresponding activation thresholds at both locations. This configuration would be a good candidate for bilateral stimulation.

#### 3.2.2. Three-dimensional ROI and ROA

**Figure 8.**
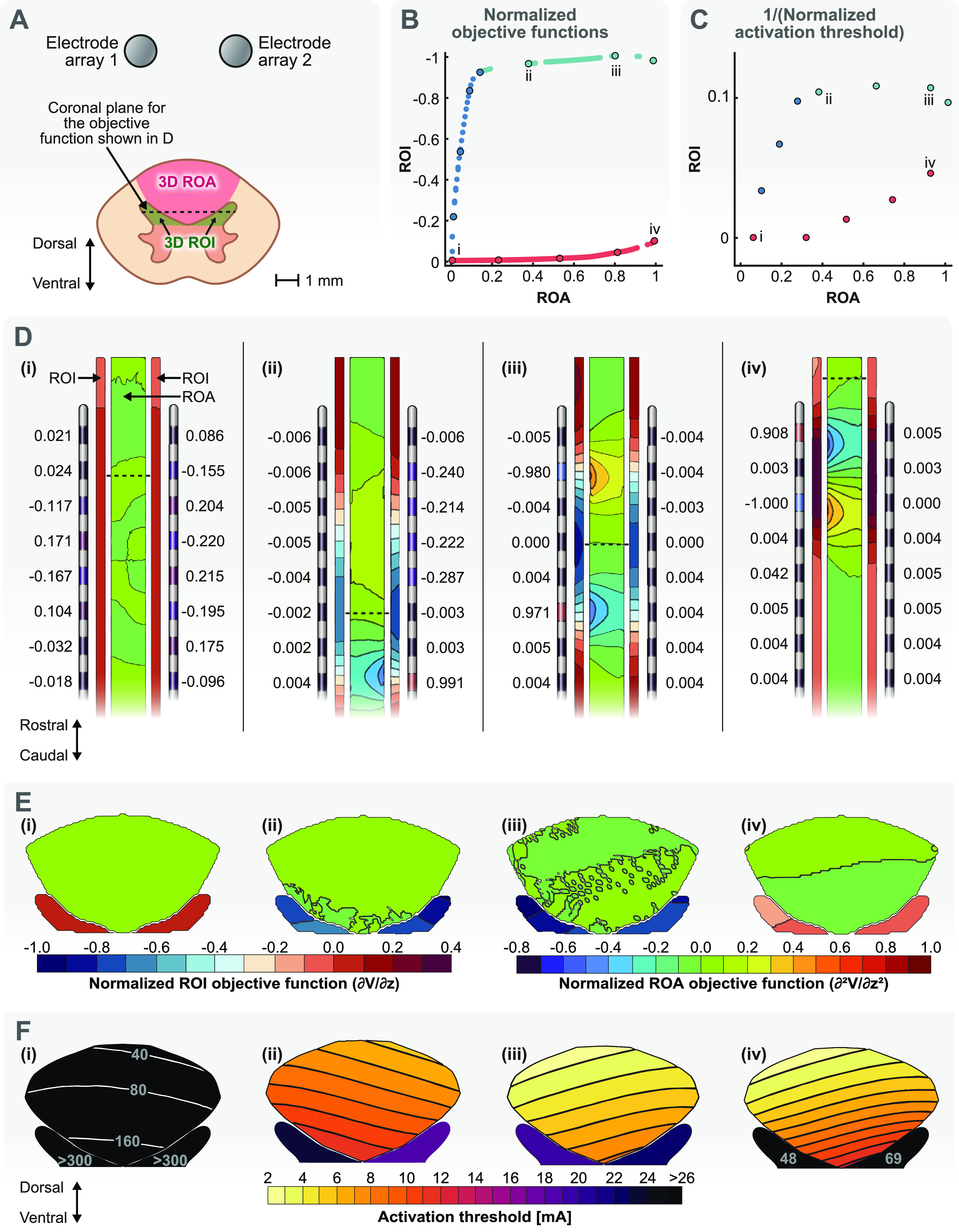
Multi-objective optimization of a three-dimensional ROI and a three-dimensional ROA. (A) The ROI is the combination of both dorsal horns. The ROA is the dorsal columns. (B) The Pareto front for multi-objective optimization. The objective function for the ROI is the negative of the derivative of the electric potential (the electric field) and the objective function for the ROA is the activating function. Each objective function was normalized using their own largest objective function. Note that large negative values are desired for the objective function in the ROI. Each point on the Pareto front represents a unique stimulation configuration. We picked several points on the Pareto front that could characterize the branches properly. We used these points to calculate the activation threshold to construct the Pareto front for the activation thresholds. We also picked four of these points on the Pareto front for further demonstration: configuration (i) represents a case in which the objective function is small for both the ROI and ROA. Configuration (ii) represents a case in which the objective function is as large as it can get in the ROI while the objective function in the ROA is still small. Configuration (iii) represents the case in which the objective function in the ROI has its largest value. Configuration (iv) represents a case in which the objective function in the ROA is close to its largest value while the objective function for the ROI is close to zero. (C) The Pareto front for activation thresholds for the selected points that characterize different branches of the Pareto front shown in (B). The activation thresholds are normalized using the smallest activation threshold detected in either the ROI or ROA. (D) Electrode configurations and the objective functions in the ROI and ROA for the points selected in B. The dashed lines show the location of the largest objective function values for the ROI (dorsal horns). (E) Contours of the objective functions in the dorsal horns and dorsal columns for the dashed lines shown in D. (F) Contours of the activation thresholds for each stimulation configuration for cells within the dorsal horn and axons within the dorsal columns (see figure 4(B)).

Finally, we considered a multi-objective optimization in which both the ROI and ROA were three-dimensional. We considered both DHs as the ROI and the entire DC as the ROA (figure 8(A)). This case also shows that optimization domains do not need to be connected in our optimization framework. The motivation for this multi-objective optimization was reports that cells in the DH can be activated with minimal activation of axons in the DCs [51–53]. If possible, such neurostimulation can provide therapeutic results without the often unwanted paresthesias associated with direct activation of DC axons (figure 1).

Figure 8(B) shows the Pareto front for this multi-objective optimization. The objective function for the ROA is again the activating function. However, the objective function for the ROI was the electric field in the rostral-caudal direction because the electric field is known to be predictive of the activation of axon terminals of local cells in the DH [13, 38]. Therefore, this optimization problem also serves as an example in which the fields that are optimized in the ROI and ROA are different. Hence, we normalized each objective function with their own maximum values in figure 7(B).

For this optimization, we did not calculate the activation thresholds for all the configurations (each point on the Pareto front shown in figure 8(B)). Instead, we found the thresholds for a limited number of points which could characterize the Pareto branches clearly. The results for the Pareto front of activation thresholds are shown in figure 8(C). Again, we plotted 1/thresholds to obtain a shape similar to the Pareto front of the objective functions (figure 8(B)). We also used the smallest activation threshold in the ROI or ROA to perform the normalization, which happened to be in the ROA. Hence, the *x*-axis values (ROA thresholds) in figure 8(C) range between 0–1 while the *y*-axis values (ROI thresholds) range approximately between 0–0.1. This result shows that the axons in the ROA are activated before the local cells in the ROI.

Next, we picked four different configurations to investigate activation of local cells in the ROI (DHs) against activation of axons in the ROA (DCs). These configurations are shown on the Pareto front of figure 7(B) (and their corresponding activation thresholds in figure 7(C)).

Previously, we showed that our optimization framework can find configurations that avoid activation in the DCs altogether (figure 6(D) or 7(D) and (E)(i)). This ability would be an attractive option here because we considered the DCs as an ROA. Configuration (i) represents one of the cases in which the objective function for the ROA is close to zero (figure 8(D)). However, figure 8(B) shows that configuration (i) also leads to a small objective function and consequently large activation thresholds in the ROI (figure 8(C)). This result shows that it is insufficient to perform a single-objective optimization to minimize activation in the ROA with the expectation that it would then allow for selective activation in the ROI. Note that for the single-objective example, a near-zero objective function (figure 6(D)) was achieved by a different set of current fractions compared to configuration (i) in figure 8(D). Yet, the current fractions for both solutions are similar in the sense that the fractions are small and contacts that face each other on the adjacent electrode have opposite polarities.

Configuration (ii) represents a logical choice for activating the ROI while not activating the ROA, because as seen in figure 8(B), the objective function of this configuration for the ROI is near its largest value while the objective function for the ROA is still close to zero. However, figures 8(C) and (F) show that even for this configuration, the axons in the ROA activate before the local cells in the ROI. Figure 8(D)(ii) shows the current fractions for this configuration and the location where the objective functions are maximum for the ROI and ROA. The current fractions for this configuration show that using a single electrode array (array 2 here) was enough to obtain the optimized results. However, figure 8(E)(ii) shows that the large objective functions are only occurring in the right DH near the electrode array 2. Therefore, the other electrode can still be useful if we wanted to formulate an optimization problem that allowed for selective stimulation between the DHs (each DH would need to be considered as a separate objective). Figure 8(F)(ii) shows that even for this optimized configuration, the axons in the deepest parts of the DC are activated before the local cells in the DH.

Configuration (iii) represents a case in which the objective function for the ROI is larger compared to configuration (ii) but at the expense of a larger objective function in the ROA. Figure 8(D)(iii) shows that only a single electrode is again necessary to achieve this result. Figure 8(F)(iii) shows that although the activation thresholds are lower in the DH, they are now also lower in the DCs. Therefore, this configuration does not really achieve the goal of activating the ROI before the ROA. This result is also evident when we compare points (ii) and (iii) on the threshold Pareto front in figure 8(C).

Finally, configuration (iv) represents a situation in which the ROA is activated before the ROI and would only be a logical choice if we switched the ROI and ROA. Figure 8(B) shows that this configuration is at a point on the Pareto front where the objective function for the ROA is at its largest when the objective function for the ROI is close to zero. In fact, this configuration is similar to single-objective optimization when the DCs were a three-dimensional ROI. For this configuration, Figure 8(F)(iv) shows that the activation thresholds are low for the DC but the local cells in the DH are activated at much larger amplitudes.

## 4. Discussion

In recent years, we have seen several technological developments in SCS [54], which includes the introduction of new waveforms, closed-loop systems, and high-density electrode arrays. The goal of all these efforts is to overcome the shortcomings of SCS. While these advances increase the potential of improving patient care, they also introduce a challenge: searching the large parameter space of stimulation settings is no longer possible during a clinical visit [11]. Moreover, the traditional method of programming, which relies on picking stimulation parameters that generate maximal overlap between stimulation-induced parasthesia and a patient’s primary pain areas, is becoming obsolete because many of the new SCS paradigms are either paresthesia-free or the onset of pain relief is not immediate [55, 56]. Hence, the need for a systematic approach to efficiently select programming settings is crucial in order to harness technological advances to improve patient care.

In this study, we presented an optimization framework that enables an efficient paradigm to choose current fractions across electrode arrays to achieve targeted SCS. In general, the utilization of optimization routines for programming SCS and other neurostimulation approaches is limited. Many optimization studies in the neurostimulation field focus on DBS [57–60] with some of them focusing on retinal prostheses [61–65]. These studies use analytic methods [57] or heuristic approaches [58].

Analytic methods have the advantage of finding an optimized solution faster, but they can be limited because of how they need to be formulated. For example, one optimization approach for SCS formulates the optimization as maximizing a field in a volume while minimizing the same field everywhere else and enables the use of eigenvectors to find the optimized solution [66]. However, this sophisticated approach has at least two limitations that should be stated. First, this approach essentially assumes that all the tissue outside of the ROI is an ROA. This assumption is not completely valid in practice and it can affect the optimized solutions that are found. Second, the field that governs activation within the ROI (e.g. the electric field) might be different from the field that governs excitation within the ROA (e.g. the electric field in another direction or the activating function). Because of these two limitations, this approach cannot be used for multi-objective optimization for SCS, which can be regarded as another limitation.

To avoid potential challenges associated with analytic methods, optimization algorithms can utilize heuristic approaches that allow for more freedom regarding how the optimization problem is formulated. For example, directly relevant to neural activation, heuristic approaches can allow for optimizing the maximum of the activating function (i.e. second order spatial derivative of the applied potential field) in a volume [58]. The possibility to optimize the maximum of a field is important because a tissue can be regarded as activated even if a small portion of the targeted area passes a threshold. Normally, finding the maximum of the activating function would not be possible using analytic methods because the maximum operator is not a differentiable function, and many analytic methods rely upon derivatives of functions. However, potential drawbacks of heuristic approaches are that they can be slow and do not necessarily converge to the true global optimum solution.

Our optimization framework is an analytic method that uses the Lagrange multipliers method. Hence, compared to heuristic algorithms, our algorithm is fast and finds all of the optimized solutions, including the global solution, provided that they exist. Yet, we are not restricted to some of the limitations of analytic methods described above. Namely, we were able to optimize the maximum of any field because we used a smooth maximum function instead of the exact maximum operator. Moreover, we are not limited in the types of fields (order of derivatives or their directions), or the dimension of the fields that are considered for optimization. Hence, we were able to extend our optimization framework to multi-objective optimization.

Many of the results produced by our algorithm resemble bipolar configurations (i.e. a cathode and anode pair). However, our results are not exact bipolar configurations because of numerical inaccuracies and approximations of exact functions in our methods (see section 2.3 for details). In fact, exact bipolar configuration can result in larger objective functions compared to the values found by our algorithm (figure 6(B)). Indeed, a simple bipolar configuration is commonly selected by experienced programmers to target axons within the DCs. However, even if we assume that a bipolar configuration can always produce adequate results, selecting proper anodes and cathodes is crucial. For example, figure 5(C) shows that our optimized configuration (that resembles a bipole) has a larger objective function in the deep DCs compared to a bipolar configuration, which is separated by one contact (similar to the configuration shown in figure 6(B)). Hence, we can argue that increased spacing between cathodes and anodes can result in higher objective functions (lower activation thresholds) in deeper regions of the DCs. Then, this argument can be used to provide general guidelines for programming based on the location of the ROIs (e.g. ROIs deeper within the DCs require increased spacing between the active electrodes). However, trying to provide general guidelines is prone to failure because patients’ needs, their unique anatomy, or the surgical outcomes cannot be generalized.

While it is true that our algorithm suggests a bipolar stimulation configuration in simple scenarios, this result is expected because a bipolar configuration was largely derived from first principles using idealized canonical models. We believe that the true utility of our optimization framework will be achieved when it is implemented in a patient-specific manner. For example, the electrodes might have non-ideal placement relative to the spinal cord (e.g. due to natural migration of electrodes over time). Factors, such as interpatient variability in anatomy and lead placement, do not affect the ability of our optimization framework to suggest proper electrode configurations for the desired targets. These factors can be accounted for by performing simulations with canonical models over a range of different anatomical parameters (e.g. dorsal CSF thickness) and/or lead placements (e.g. placement relative to the spinal cord midline) and utilizing lookup tables of optimized parameters for the situation that best matches the conditions for the individual patient. Furthermore, these sources of intrapatient variability can be accounted for in patient-specific computational models [14, 34, 67, 68] in which we can then apply our optimization framework to determine optimal patient-specific stimulation parameters. It is also important to recognize that the application of our optimization framework is not limited to chronic pain management using SCS. SCS has recently been shown to improve rehabilitation of patients after spinal cord injury [69, 70]. Patient-specific modeling has proven to be an important part of this application [71] and it can benefit from optimization for better clinical results.

Moreover, targeting multiple ROIs while avoiding ROAs can hinder the ability of even an experienced programmer to find proper stimulation configurations because simple configurations (e.g. bipole, guarded cathode) may prove inadequate for such scenarios. This common situation is where our framework can provide a solution for these challenges by formulating them as multi-objective optimization problems. Our results in figures 7 and 8 demonstrate the ability of our optimization framework to achieve these goals.

In figure 7, we considered two axons (each near an electrode array) as targets. One of the results suggested by our algorithm (configuration (iii)) resembles a bipolar configuration. This configuration on the Pareto fronts corresponds to a point on which targeting the axon near the electrode array 1 is the only goal. Hence, not surprisingly but correctly, our algorithm suggested a bipolar configuration on the electrode array near the target as a human programmer would have done. However, we can consider a case in which we want to target both axons equally (e.g. to relieve bilateral pain). A bipolar configuration cannot achieve this goal, but a configuration suggested by our algorithm (configuration (iv)) is capable of this task (compare figures 7(iii) and (iv)). Similarly, we can consider a case in which we want to only target one of the axons and avoid activating the other axon altogether. Again, a bipolar configuration is incapable of this task while our optimization framework can provide a configuration (configuration (ii)) for this task (compare figures 7(ii) and (iii)).

Targeting ROIs while avoiding ROAs is a topic that has not received as much attention in SCS as it has in other applications, such as DBS [59]. However, some recent studies [51, 72] have suggested that directly targeting cells in the DH can create pain relief while avoiding concomitant paresthesias associated with the activation of large-diameter fibers in the DCs that can be experienced as excessive or uncomfortable. Hence, the DHs can be regarded as ROIs and the DCs as a ROA. We used our optimization framework to investigate the trade-off between activation of the ROI and ROA (figure 8). For our model geometry, our results suggest that direct activation of cells in the DH is not possible before activation of cells in the DCs. However, our optimization algorithm was able to suggest a nontrivial configuration (configuration (ii)) that leads to lower activation thresholds for cells in the DH compared to a configuration that resembles a bipolar configuration (configuration (iv)). As introduced in figure 1, this same multi-objective approach could be implemented for a ROI for activating DC fibers to provide pain relief with a ROA corresponding to dorsal root fibers whose activation is associated with unwanted side effects [5, 73].

With regards to clinical implementation of our optimization framework, the novel configurations obtained from our optimization framework require selecting current fractions across multiple electrodes. In practice, manual adjustment of the current levels across individual electrodes would lead to long programming sessions with current commercial systems. Therefore, our optimization framework would ideally be integrated with a stimulation system to allow a programmer to directly choose ROIs and the current fractions would be applied automatically across the electrode arrays. Also, in the case of multi-objective optimization, the programmer would be presented with a Pareto front from which they could choose specific points to try and the current fractions corresponding to the individual point on the Pareto front would be implemented automatically.

Our optimization framework has some potential limitations that should be considered. First, our framework is based on the principle of superposition. Hence, our optimization framework can only be applied to current-controlled stimulation systems and is not applicable to voltage-controlled stimulators. However, most clinical neurostimulation systems utilize current-controlled stimulation. Second, the input to our optimization framework are the FEM solutions for the volume conductor model, which can be computationally expensive. However, this is a step that is conducted once in advance and before the optimization step. Therefore, it does not affect the optimization process itself. Moreover, if it is necessary to reduce the computational demands, the FEM model can be reduced in complexity and/or replaced by less accurate simplified approximations, such as the model used in our illustrative example (figure 3). Third, we have only focused on the spatial optimization in this study and neglected temporal features of neurostimulation. However, with fields being optimized, a logical next step would be to optimize the temporal features of the stimulation (e.g. pulse width, pulse frequency). Finally, this manuscript only describes our optimization methodology and future research will be critical to confirm the translatability of this approach for providing targeted SCS in patients.

## 5. Conclusion

In an attempt to improve the clinical outcomes associated with SCS to treat chronic pain, clinical neurostimulation systems have advanced to include a wide range of stimulation parameters, high electrode counts, and systems that can fractionate stimulus amplitudes across individual electrodes. These innovations correspond to a dramatic increase in the number of possible stimulations settings that cannot be explored within the context of standard clinical programming procedures. Furthermore, evidence suggests that emerging SCS paradigms may have distinct sites of action and there is corresponding interest in the ability to selectively target specific neural structures. To facilitate efficient exploration of the stimulation parameter space, we described a novel optimization framework for targeted stimulation. Our approach has the advantage that it utilizes the speed of analytic methods and the ability to find global optimums for both single- and multi-objective optimizations. Furthermore, our optimization framework can be implemented in a patient-specific approach to account for sources of interpatient variability (e.g. anatomy, lead placement) and expanded to additional neuromodulation technologies (e.g. DBS). As clinical neurostimulation systems continue to increase in complexity, grow in popularity, and expand to new indications, we believe that optimization frameworks like ours will be a critical component to maintain the tractability and efficacy of these systems.

## Data Availability

The data cannot be made publicly available upon publication because they contain commercially sensitive information. The data that support the findings of this study are available upon reasonable request from the authors.

## References

[1] Sdrulla A D, Guan Y, Raja S N (2018). Spinal cord stimulation: clinical efficacy and potential mechanisms. Pain Pract..

[2] Adil S M (2021). Impact of spinal cord stimulation on opioid dose reduction: a nationwide analysis. Neurosurgery.

[3] Taylor R S, Desai M J, Rigoard P, Taylor R J (2014). Predictors of pain relief following spinal cord stimulation in chronic back and leg pain and failed back surgery syndrome: a systematic review and meta‐regression analysis. Pain Pract..

[4] Rigoard P (2022). The challenge of converting “failed spinal cord stimulation syndrome” back to clinical success, using SCS reprogramming as salvage therapy, through neurostimulation adapters combined with 3D-computerized pain mapping assessment: a real life retrospective study. J. Clin. Med..

[5] Titus N D, Gilbert J E, Grill W M (2021). Biophysics and mechanisms of spinal cord stimulation for chronic pain. Handbook of Neuroengineering.

[6] Jensen M P, Brownstone R M (2019). Mechanisms of spinal cord stimulation for the treatment of pain: still in the dark after 50 years. Eur. J. Pain.

[7] Kumar K, Caraway D L, Rizvi S, Bishop S (2014). Current challenges in spinal cord stimulation. Neuromodulation.

[8] Sheldon B, Staudt M D, Williams L, Harland T A, Pilitsis J G (2021). Spinal cord stimulation programming: a crash course. Neurosurg. Rev..

[9] Gordon A T, Zou S P, Kim Y, Gharibo C (2007). Challenges to setting spinal cord stimulator parameters during intraoperative testing: factors affecting coverage of low back and leg pain. Neuromodulation.

[10] Gharibo C, Laux G, Forzani B R, Sellars C, Kim E, Zou S (2014). State of the field survey: spinal cord stimulator use by academic pain medicine practices. Pain Med..

[11] Moffitt M A, Lee D C, Bradley K, Greenbaum E, Zhou D (2009). Spinal cord stimulation: engineering approaches to clinical and physiological challenges. Implantable Neural Prostheses 1. Biological and Medical Physics, Biomedical Engineering.

[12] Veizi E (2017). Spinal cord stimulation (SCS) with anatomically guided (3D) neural targeting shows superior chronic axial low back pain relief compared to traditional SCS—LUMINA study. Pain Med..

[13] Metzger C S, Hammond M B, Pyles S T, Washabaugh E P, Waghmarae R, Berg A P, North J M, Pei Y, Jain R (2020). Pain relief outcomes using an SCS device capable of delivering combination therapy with advanced waveforms and field shapes. Expert Rev. Med. Devices.

[14] Lempka S F, Zander H J, Anaya C J, Wyant A, Ozinga Iv J G, Machado A G (2020). Patient-specific analysis of neural activation during spinal cord stimulation for pain. Neuromodulation.

[15] Chakraborty D, Truong D Q, Bikson M, Kaphzan H (2018). Neuromodulation of axon terminals. Cereb. Cortex.

[16] Hause L (1975). A mathematical model for transmembrane potentials secondary to extracellular fields. Electroanesthesia.

[17] Hentall I D (1985). The membrane potential along an ideal axon in a radial electric field. Brain Res..

[18] Rattay F (1986). Analysis of models for external stimulation of axons. IEEE Trans. Biomed. Eng..

[19] McNeal D R (1976). Analysis of a model for excitation of myelinated nerve. IEEE Trans. Biomed. Eng..

[20] Butson C R, McIntyre C C (2008). Current steering to control the volume of tissue activated during deep brain stimulation. Brain Stimul..

[21] Plonsey R, Heppner D B (1967). Considerations of quasi-stationarity in electrophysiological systems. Bull Math Biophys.

[22] Lee D, Gillespie E, Bradley K (2011). Dorsal column steerability with dual parallel leads using dedicated power sources: a computational model. J. Vis. Exp..

[23] Bertsekas D P (2014). Constrained Optimization and Lagrange Multiplier Methods.

[24] Spring D (1985). On the second derivative test for constrained local extrema. Am. Math. Mon..

[25] Collette Y, Siarry P (2004). Multiobjective Optimization.

[26] Mirzakhalili E, Barra B, Capogrosso M, Lempka S F (2020). Biophysics of temporal interference stimulation. Cell Syst..

[27] Foster K R, Schwan H P (1989). Dielectric properties of tissues and biological materials: a critical review. Crit. Rev. Biomed. Eng..

[28] Capogrosso M, Wenger N, Raspopovic S, Musienko P, Beauparlant J, Luciani L B, Courtine G, Micera S (2013). A computational model for epidural electrical stimulation of spinal sensorimotor circuits. J. Neurosci..

[29] Ranck J B Jr, BeMent S L (1965). The specific impedance of the dorsal columns of cat: an anisotropic medium. Exp. Neurol..

[30] Zander H J, Graham R D, Anaya C J, Lempka S F (2020). Anatomical and technical factors affecting the neural response to epidural spinal cord stimulation. J. Neural Eng..

[31] Anaya C J, Zander H J, Graham R D, Sankarasubramanian V, Lempka S F (2020). Evoked potentials recorded from the spinal cord during neurostimulation for pain: a computational modeling study. Neuromodulation.

[32] Geddes L A, Baker L E (1967). The specific resistance of biological material–a compendium of data for the biomedical engineer and physiologist. Med. Biol. Eng..

[33] Lempka S F, McIntyre C C, Kilgore K L, Machado A G (2015). Computational analysis of kilohertz frequency spinal cord stimulation for chronic pain management. Anesthesiology.

[34] Howell B, Lad S P, Grill W M (2014). Evaluation of intradural stimulation efficiency and selectivity in a computational model of spinal cord stimulation. PLoS One.

[35] Gabriel S, Lau R W, Gabriel C (1996). The dielectric properties of biological tissues: II. Measurements in the frequency range 10 Hz to 20 GHz. Phys. Med. Biol..

[36] Grill W M, Mortimer J T (1994). Electrical properties of implant encapsulation tissue. Ann. Biomed. Eng..

[37] McIntyre C C, Richardson A G, Grill W M (2002). Modeling the excitability of mammalian nerve fibers: influence of afterpotentials on the recovery cycle. J. Neurophysiol..

[38] Rogers E R, Zander H J, Lempka S F (2022). Neural recruitment during conventional, burst, and 10-kHz spinal cord stimulation for pain. J. Pain.

[39] Luz L L, Szucs P, Pinho R, Safronov B V (2010). Monosynaptic excitatory inputs to spinal lamina I anterolateral-tract-projecting neurons from neighbouring lamina I neurons. J. Physiol..

[40] Aberra A S, Peterchev A V, Grill W M (2018). Biophysically realistic neuron models for simulation of cortical stimulation. J. Neural Eng..

[41] Zhang T C, Janik J J, Grill W M (2014). Modeling effects of spinal cord stimulation on wide-dynamic range dorsal horn neurons: influence of stimulation frequency and GABAergic inhibition. J. Neurophysiol..

[42] Wolff M, Vogel W, Safronov B V (1998). Uneven distribution of K+ channels in soma, axon and dendrites of rat spinal neurones: functional role of the soma in generation of action potentials. J. Physiol..

[43] Melnick I V, Santos S F, Szokol K, Szucs P, Safronov B V (2004). Ionic basis of tonic firing in spinal substantia gelatinosa neurons of rat. J. Neurophysiol..

[44] Gentet L J, Stuart G J, Clements J D (2000). Direct measurement of specific membrane capacitance in neurons. Biophys. J..

[45] Ruscheweyh R, Sandkuhler J (2002). Lamina-specific membrane and discharge properties of rat spinal dorsal horn neurones in vitro. J. Physiol..

[46] McIntyre C C, Grill W M (2000). Selective microstimulation of central nervous system neurons. Ann. Biomed. Eng..

[47] Hines M L, Carnevale N T (1997). The NEURON simulation environment. Neural Comput..

[48] Hines M L, Davison A P, Muller E (2009). NEURON and Python. Front. Neuroinform..

[49] Rattay F (1990). Electrical Nerve Stimulation.

[50] Billot M (2020). Comparison of conventional, burst and high-frequency spinal cord stimulation on pain relief in refractory failed back surgery syndrome patients: study protocol for a prospective randomized double-blinded cross-over trial (MULTIWAVE study). Trials.

[51] Li S, Zhang T, Zhu C, Farber J, Gu W, Esteller R, Moffitt M, Linderoth B, Foreman R D (2019). Abstract #44: computational and immunohistochemical evidence of direct dorsal horn modulation by sub-perception spinal cord stimulation. Brain Stimul..

[52] Lee K Y, Lee D, Wang D, Kagan Z B, Bradley K (2022). Simultaneous 10 kHz and 40 Hz spinal cord stimulation increases dorsal horn inhibitory interneuron activity. Neurosci. Lett..

[53] Lee K Y, Lee D, Kagan Z B, Wang D, Bradley K (2021). Differential modulation of dorsal horn neurons by various spinal cord stimulation strategies. Biomedicines.

[54] Lempka S F, Patil P G (2018). Innovations in spinal cord stimulation for pain. Curr. Opin. Biomed. Eng..

[55] Chakravarthy K, Richter H, Christo P J, Williams K, Guan Y (2018). Spinal cord stimulation for treating chronic pain: reviewing preclinical and clinical data on paresthesia-free high-frequency therapy. Neuromodulation.

[56] Linderoth B, Foreman R D (2017). Conventional and novel spinal stimulation algorithms: hypothetical mechanisms of action and comments on outcomes. Neuromodulation.

[57] Anderson D N, Osting B, Vorwerk J, Dorval A D, Butson C R (2018). Optimized programming algorithm for cylindrical and directional deep brain stimulation electrodes. J. Neural Eng..

[58] Pena E, Zhang S, Deyo S, Xiao Y, Johnson M D (2017). Particle swarm optimization for programming deep brain stimulation arrays. J. Neural Eng..

[59] Connolly M J, Cole E R, Isbaine F, de Hemptinne C, Starr P A, Willie J T, Gross R E, Miocinovic S (2021). Multi-objective data-driven optimization for improving deep brain stimulation in Parkinson’s disease. J. Neural Eng..

[60] Malekmohammadi M (2022). Automated optimization of deep brain stimulation parameters for modulating neuroimaging-based targets. J. Neural Eng..

[61] Shah N P, Madugula S, Grosberg L, Mena G, Tandon P, Hottowy P, Sher A, Litke A, Mitra S, Chichilnisky E J (2019). Optimization of electrical stimulation for a high-fidelity artificial retina. 9th Int. IEEE/EMBS Conf. on Neural Engineering (NER).

[62] Haji Ghaffari D, Akwaboah A D, Mirzakhalili E, Weiland J D (2021). Real-time optimization of retinal ganglion cell spatial activity in response to epiretinal stimulation. IEEE Trans. Neural Syst. Rehabil. Eng..

[63] Abouelseoud G, Abouelseoud Y, Shoukry A, Ismail N, Mekky J (2023). A mixed integer linear programming framework for improving cortical vision prosthesis designs. Biomed. Signal Process. Control.

[64] Fauvel T, Chalk M (2022). Human-in-the-loop optimization of visual prosthetic stimulation. J. Neural Eng..

[65] Ghaffari D H, Chang Y C, Mirzakhalili E, Weiland J D (2021). Closed-loop optimization of retinal ganglion cell responses to epiretinal stimulation: a computational study. 10th Int. IEEE/EMBS Conf. on Neural Engineering (NER).

[66] Mishra L N, Kulkarni G, Gadgil M (2023). A novel current steering method for targeted spinal cord stimulation. Front. Pain Res..

[67] Solanes C, Dura J L, Angeles Canos M, De Andres J, Marti-Bonmati L, Saiz J (2021). 3D patient-specific spinal cord computational model for SCS management: potential clinical applications. J. Neural Eng..

[68] Liang L, Damiani A, Del Brocco M, Rogers E R, Jantz M K, Fisher L E, Gaunt R A, Capogrosso M, Lempka S F, Pirondini E (2022). A systematic review of computational models for the design of spinal cord stimulation therapies: from neural circuits to patient-specific simulations. J. Physiol..

[69] Wagner F B (2018). Targeted neurotechnology restores walking in humans with spinal cord injury. Nature.

[70] Powell M P (2023). Epidural stimulation of the cervical spinal cord for post-stroke upper-limb paresis. Nat. Med..

[71] Rowald A (2022). Activity-dependent spinal cord neuromodulation rapidly restores trunk and leg motor functions after complete paralysis. Nat. Med..

[72] Russo M A, Volschenk W, Bailey D, Santarelli D M, Holliday E, Barker D, Dizon J, Graham B (2023). A novel, paresthesia-free spinal cord stimulation waveform for chronic neuropathic low back pain: six-month results of a prospective, single-arm, dose-response study. Neuromodulation.

[73] Struijk J J, Holsheimer J, Boom H B (1993). Excitation of dorsal root fibers in spinal cord stimulation: a theoretical study. IEEE Trans. Biomed. Eng..

